# Hydrodynamic Modeling Coupled with Long-term Field Data Provide Evidence for Suppression of Phytoplankton by Invasive Clams and Freshwater Exports in the San Francisco Estuary

**DOI:** 10.1007/s00267-019-01159-6

**Published:** 2019-04-03

**Authors:** Bruce G. Hammock, Samuel P. Moose, Samuel Sandoval Solis, Erfan Goharian, Swee J. Teh

**Affiliations:** 1 Aquatic Health Program, Department of Anatomy, Physiology, and Cell Biology, School of Veterinary Medicine, University of California, 1089 Veterinary Medicine Drive, VetMed 3B, Davis, CA 95616 USA; 2 Department of Land, Air and Water Resources, University of California at Davis, Davis, CA 95616 USA; 3 Civil and Environmental Engineering Department, University of South Carolina, C113B, 300 Main St., Columbia, SC 29208 USA

**Keywords:** Residence time, Particle tracking, Chlorophyll a, Productivity, *Potamocorbula amurensis*

## Abstract

The San Francisco Estuary (California, USA) had abundant pelagic fish in the late 1960s, but has few pelagic fish today. A primary cause for this decline in fish is thought to be a trophic cascade, triggered by declining phytoplankton. Here, we describe the changes in pelagic community structure of the San Francisco Estuary. Then, we examine whether changes in hydrodynamics due to freshwater exports, which increased exponentially beginning in 1967, in addition to the 1986 invasion by the clam *Potamocorbula amurensis*, explain the phytoplankton loss. Hydrodynamic variables were reconstructed back to 1956 using statistical models fit to, and cross-validated against, output from a hydrodynamic model. Then, we regressed mean summer/fall chlorophyll a—the season with the largest phytoplankton decline—against the reconstructed hydrodynamic variables and the presence/absence of *P. amurensis* for 1969–2014. The regression model, which explained 78% of the interannual variation in chlorophyll a, was then used to quantify the influence of *P. amurensis* and exports on chlorophyll a. Based on monitoring data, chlorophyll a declined 22-fold from 1969–2014, zooplankton declined 32-fold from 1972–2014, and pelagic fish declined 92-fold from 1968–2014. Averaged over 1990–2014, the chlorophyll a model ascribed an 88% decline in chlorophyll a to *P. amurensis*, a 74% decline to exports (at minimum), and a 97% decline to the combined influence of *P. amurensis* and exports (at minimum). Thus, the decline in pelagic productivity in the San Francisco Estuary has occurred largely due to the combined impacts of the *P. amurensis* invasion and increased freshwater exports.

## Introduction

Estuaries rank with agroecosystems and tropical rain forests as the most productive ecosystems in the world (Hopkinson and Smith 2005). Rivers slow and broaden as they encounter seawater, expanding the photic zone and increasing residence time—the time a water parcel takes to move through a water body (Monsen et al. 2002). An expanded photic zone and high residence time provide phytoplankton with time and energy to reproduce. Estuaries have naturally high nutrient concentrations due to their basal locations in watersheds, mobilization of nutrients from the benthos by tidal-energy, and nutrient upwelling from adjacent marine ecosystems (Odum 1967; Smith and Hollibaugh 1997; Hopkinson and Smith 2005). Anthropogenic nutrient inputs, most notably nitrogen from wastewater treatment plants and agricultural runoff, are also powerful drivers of estuarine productivity (Anderson et al. 2002; Whitall et al. 2007). These inputs often tip estuaries into eutrophic states, the most serious threat to estuarine ecosystems globally (Howarth et al. 2000; Anderson et al. 2002; Hopkinson and Smith 2005; Kemp et al. 2005). In the Chesapeake Bay for example, nutrient enrichment has elevated phytoplankton densities for over 100 years (Kemp et al. 2005). Consequently, severe, recurring periods of benthic hypoxia, first noted in the 1950s, have caused declines in macrophytes and benthic macroinfauna (Kemp et al. 2005).

**Fig. 1:**
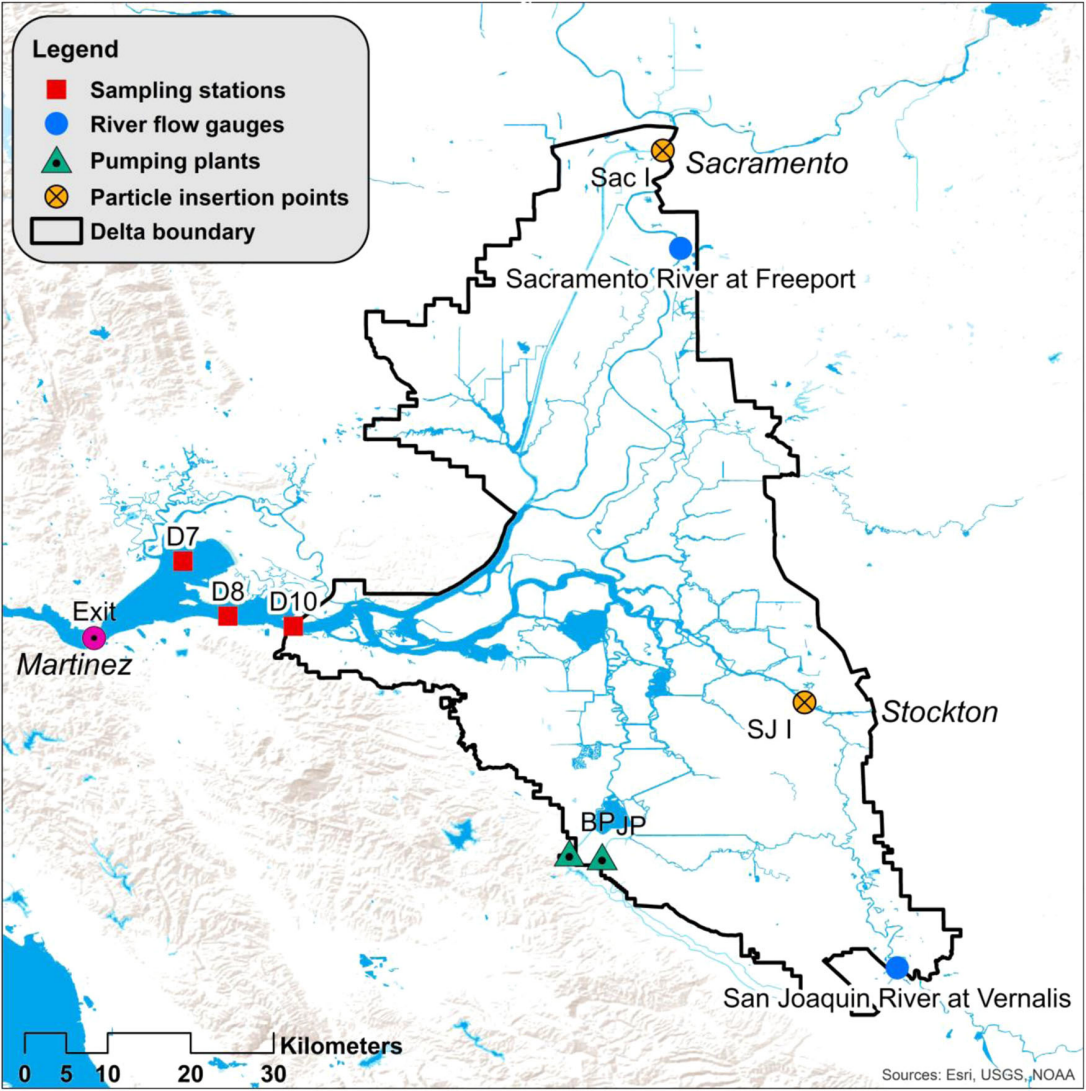
Map of the Sacramento San Joaquin Delta and SFE. The black line denotes the Delta, which meets saltwater from San Francisco Bay in the SFE. *BP* is the state Harvey Banks Pumping Plant, part of the State Water Project (SWP), *JP* is the federal Bill Jones Pumping Plant, part of the Central Valley Project (CVP). *Sac I* is the particle insertion point on the Sacramento River, *SJ I* is the same on the San Joaquin River, and *Exit* denotes the Western boundary of the estuary for the purposes of DSM2 particle tracking. D10, D8, and D7 represent the three approximate locations with continuous monitoring of water quality, fish, and zooplankton (exact locations in Table S6), and are the sites for which chlorophyll a was modeled. Benthic organisms are monitored at D7, not at D8, and D10

Given the global prevalence of estuarine eutrophication, the San Francisco Estuary (SFE) presents a surprising counter-example. It is formed by the confluence of the Sacramento and San Joaquin rivers and the Pacific Ocean in central California, USA. The SFE is highly altered. An estimated 97% of its tidal wetlands were drained in the 19th and early 20th centuries (Whipple et al. 2012), it is highly invaded by exotic species (Cohen and Carlton 1998), and extensively channelized (Nichols et al. 1986). Two large pumping plants—part of the federally operated Central Valley Project (CVP) and the California operated State Water Project (SWP)—export fresh water from the southern Delta (landward of the SFE) to supply cities and farms to the south, while most of the fresh water enters from the north via the Sacramento River (Kimmerer 2004; Fig. 1). Nutrient concentrations in the SFE are elevated, mainly due to discharge from several large wastewater treatment plants and agricultural runoff (Jassby et al. 2002; Kimmerer et al. 2012; Dahm et al. 2016). However, while the estuary was productive as recently as the early 1980s, it is currently one of the least productive estuaries in the world (i.e., <100 g C m^−2^ year^−1^; Cloern et al. 2014; Wilkerson and Dugdale 2016). The low pelagic productivity of the SFE is considered a primary cause for the low abundance of several resident fish species (Sommer et al. 2007), including the imperiled Delta Smelt (Feyrer et al. 2003; Sommer et al. 2007; Hammock et al. 2017; Hamilton and Murphy 2018).

Despite intensive research and decades of monitoring (e.g., Kimmerer 2004; Dugdale et al. 2007; Dahm et al. 2016), the causes for the low productivity of the SFE remain unclear. Researchers have proposed five hypotheses to explain the low abundance of phytoplankton: (1) grazing by the invasive clam *Potamocorbula amurensis* (Alpine and Cloern 1992), (2) ammonia inhibition of diatom growth (Parker et al. 2012; Wilkerson and Dugdale 2016), (3) phosphorus limitation (Van Nieuwenhuyse 2007), (4) elevated nitrogen, resulting in an unsuitably high nitrogen to phosphorus ratio (Glibert 2010; Glibert et al. 2011), and (5) freshwater exports from the south Delta, which removes phytoplankton from the estuary and may reduce residence time (Jassby and Powell 1994; Arthur et al. 1996). Of these hypotheses, grazing by *P. amurensis* is the most widely accepted (e.g., Jassby et al. 2002; Feyrer et al. 2003; Kimmerer 2004; Kimmerer 2006; Cloern and Jassby 2012; Dahm et al. 2016). The evidence for the grazing hypothesis is two-fold. First, mean chlorophyll a decreased in Suisun Bay—the epicenter of the *P. amurensis* invasion—from 11 µg L^−1^ before the invasion (1975–1986) to 2.2 µg L^−1^ after the invasion (1987–2010; Cloern and Jassby 2012). Second, filtration rates by *P. amurensis* in Suisun Bay are estimated to exceed local phytoplankton production (Thompson 2005). However, by the time *P. amurensis* invaded in 1986, much of the primary and secondary pelagic productivity had already been lost, simultaneously from both fresh and brackish regions (Cloern and Jassby 2012; Hammock et al. 2017), indicating that *P. amurensis* is not the only cause for the loss of pelagic productivity. The three nutrient hypotheses are controversial. Nutrient levels in the SFE are considered replete (Jassby et al. 2002; Cloern et al. 2007; Kimmerer et al. 2012), and high nitrogen generally promotes algal growth (e.g., Anderson et al. 2002; Whitall et al. 2007). However, others have suggested that high nitrogen/ammonia suppresses algal growth in the SFE (e.g., Glibert et al. 2011; Parker et al. 2012), and Van Nieuwenhuyse (2007) suggests that phosphorus may be limiting. The freshwater export hypothesis (Jassby and Powell 1994) is relatively unstudied.

**Fig. 2:**
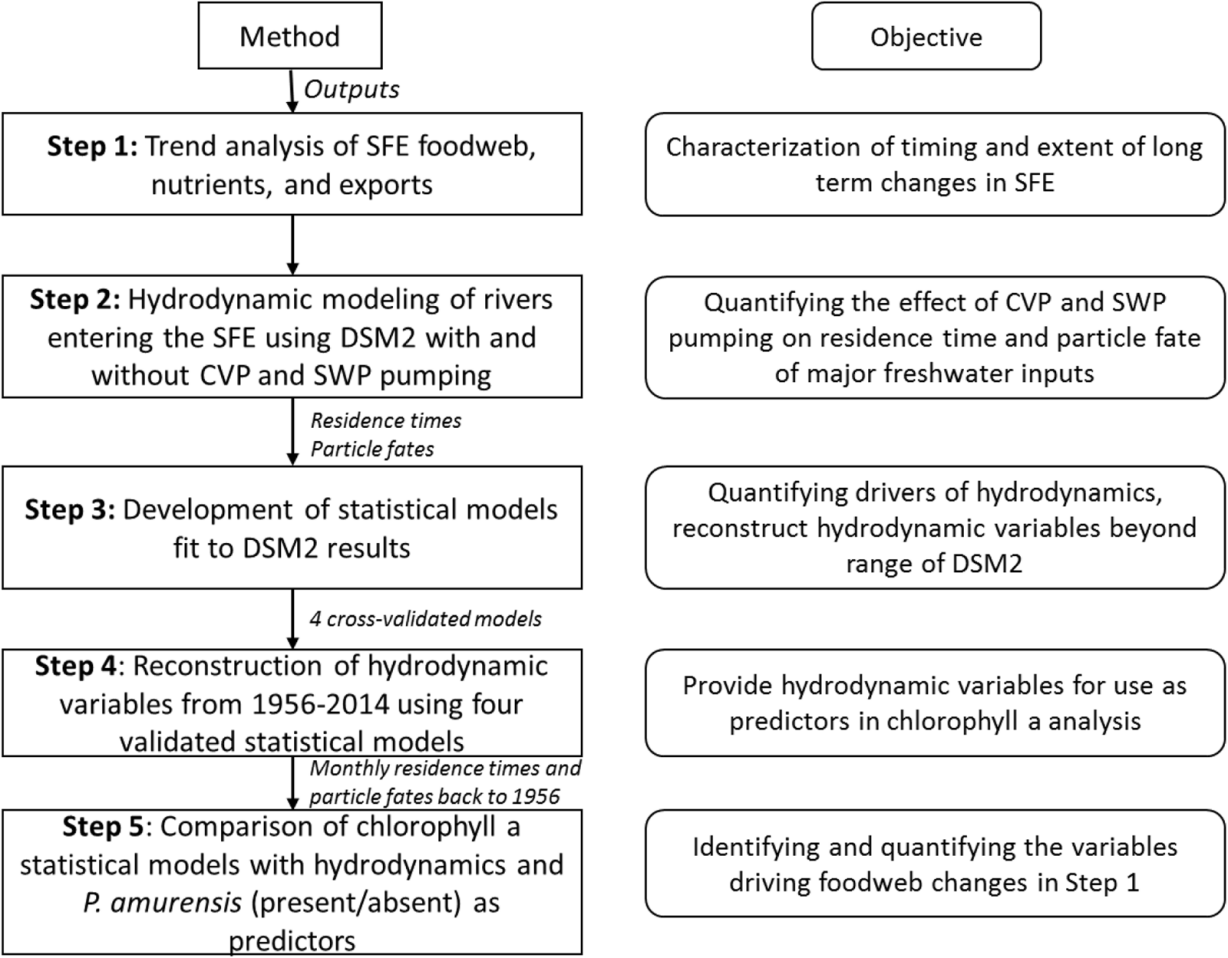
Flow diagram of the analysis

Here, we describe the changes in the food web (Fig. 2, Step 1) and then use a series of four modeling steps to examine the influence of exports on SFE phytoplankton productivity (Hypothesis 5), while accounting for the grazing of *P. amurensis* (Hypothesis 1; Fig. 2, Steps 2–5). Hydrodynamic variables were reconstructed back to 1956 using statistical models fit to, and cross-validated against, output from the hydrodynamic computer model Delta Simulation Model II (DSM2). This step was necessary because the version of the computer model we used does not extend to periods before 1989, whereas exports began increasing exponentially in 1967. Finally, we regressed mean summer/fall chlorophyll a—the season with the largest phytoplankton decline—against the reconstructed hydrodynamic variables and the presence/absence of *P. amurensis* for 1969–2014.

## Materials and Methods

### Analysis Summary

Our analysis consisted of five steps (Fig. 2) which we briefly describe here, in subsequent sections, and in the supplement. In Step 1 (Fig. 2) we used monitoring data collected by state and federal agencies to characterize the timing and extent of the long-term changes at three monitoring locations in the SFE (D7, D8, and D10; Fig. 1) in terms of six variables: chlorophyll a (µg L^−1^), meso-zooplankton (individuals m^−3^), pelagic fish (individuals trawl^−1^), invasive clams (individuals m^−2^), orthophosphate (mg L^−1^), and dissolved inorganic nitrogen (mg L^−1^; details in supplementary material, *Characterizing decadal trends*). In Step 2 (Fig. 2) we used DSM2 (V8.1), a publically available, hydrodynamic computer model of the Sacramento-San Joaquin Delta/SFE developed by the California Department of Water Resources (DWR), to quantify the combined influence of CVP and SWP exports on the two major freshwater inputs to the SFE, the Sacramento and San Joaquin rivers (DWR 2018a, b; details in supplementary material, *DSM2*). Simulations were run of neutrally buoyant particles released monthly on each river, both in terms of how long the particles spend in the SFE (i.e., a proxy for residence time), and particle fate. Particle fates included loss to agricultural diversions (agricultural diversions occur throughout the Delta, the estimates of which were extracted from DSM2), CVP and SWP exports (the combination is an estimate for loss of freshwater from the SFE), and exiting the SFE (i.e., movement past Martinez toward the Pacific Ocean; Fig. 1). We ran these simulations over six 15-month periods between 1989 and 2012 using DSM2, with the CVP and SWP pumps set either to their historical export rates (observed), or with the CVP and SWP pumps set to zero. Periods of both extremely high, normal, and extremely low flow (Sacramento River at Freeport plus San Joaquin river at Vernalis) were included to capture the range of possible hydrodynamic conditions, and included water years 1991, 1996, 1998, 1999, 2005, and 2009, and the three months following each water year (Oct, Nov, Dec). In Step 3 (Fig. 2) we fit statistical models to 2/3 of the monthly residence time and particle fate results, compared them, and selected the best model in each of the four categories (residence time and particle fate for both rivers). Predictor variables included river flow, agricultural diversions, CVP and SWP exports, and several interactions. The top model in each category was then cross-validated against the remaining 1/3 of the DSM2 results (see model cross-validation below for details). In Step 4 (Fig. 2), using each of the four top-ranked models and publically available data on exports, flow, and agricultural diversions as predictors, we reconstructed monthly residence times and particle fates for both rivers back to 1956, well before water exports began increasing exponentially in 1967 when the SWP pumping plant came online (the CVP has operated since the early 1950s). Finally, in Step 5 (Fig. 2) we fit a series of statistical models to predict chlorophyll a measurements in the SFE, using the reconstructed hydrodynamic variables and the presence/absence of *P. amurensis* as predictors. The top-ranked model was then used to quantify and compare the influence of CVP plus SWP exports and *P. amurensis* on chlorophyll a. Predictions were made under four scenarios: without *P. amurensis* and at low freshwater export levels (i.e., actual export levels during the drought year of 1977), at 1977 export levels but with *P. amurensis*, at observed (actual) export levels without *P. amurensis*, and at observed export levels and with *P. amurensis*.

### Residence Time and Particle Fate (DSM2; Step. 2)

DSM2 includes a one-dimensional mathematical module for dynamic simulation of one-dimensional hydraulics (stages, flows, velocities; i.e., HYDRO), a water quality module (mass transport processes for conservative and non-conservative constituents; i.e., QUAL), and a module for 3-D tracking of neutrally buoyant particles that runs based on HYDRO simulations (i.e., PTM) (Fig. 2). See supplemental material (*DSM2*) and two websites with additional information, including documentation, calibration, input and historic data for the model (DWR 2018a, b).

Using HYDRO, simulations were run under two conditions: with the CVP and SWP pumps operating at their historical levels of water export, or with the CVP and SWP pumps set to zero exports. Simulations were run monthly for 1991, 1996, 1998, 1999, 2005, and 2009 water years. 1991 and 1998 were the lowest and highest outflow water years (Sacramento plus San Joaquin river flow) during the years that were available for simulation in DSM2 (1989–2012), and the other four years were selected haphazardly. In addition to the 12 months of each water year, we extended simulations through Oct, Nov, and Dec of the following water year because the fall period was especially of interest. It is during fall that the Sacramento and San Joaquin river flows are lowest (Fig. S19), so the CVP and SWP pumps may exert more influence on hydrodynamics. In addition, summer/fall is the period during which phytoplankton density has declined most (Fig. S20), and is the season with the earliest available chlorophyll a measurements (Alpine and Cloern 1992; Jassby and Powell 1994; Thompson 2005).

Using PTM, 1000 neutrally buoyant particles were inserted (virtually) at one of two locations: near Sacramento on the Sacramento River (DSM2 node 331, Fig. 1 ‘Sac I’) or near Stockton on the San Joaquin River (node 21, Fig. 1 ‘SJ I’). Insertions were made with the CVP and SWP pumps either on or off based on the HYDRO simulations, such that particles were tracked under four scenarios: Particles released on the Sacramento River with the pumps on and off, and particles released on the San Joaquin River with the pumps on and off. Simulations began on the 15th of each month and were allowed to run for one year. Each particle was tracked individually by the model, and the number of days it took for 10–90% (at 10% increments) of particles to exit the water body was registered. DSM2 also quantified the fate of the particles released, which we treated as a proxy for water fate in subsequent analyses. Particles were considered to have exited the SFE once they passed Martinez (i.e., particles heading toward San Francisco Bay), entered the CVP or SWP pumps, or were diverted for agriculture (Fig. 1). Typically, for residence time studies some percentage of particles exiting is selected and defined as residence time, often 66.7% (e.g., Liu et al. 2011). Because we were interested in the effect of CVP and SWP pumping on an average unit of water, we defined residence time as the average time a particle spent in the Delta/SFE (i.e., residence times were averaged across all percentages of particles remaining).

### Development of Statistical Models Fit to DSM2 Results, Model Cross-validation (Step 3)

Statistical models were fit to the residence time and particle fate data for two purposes (Fig. 2). The first was to identify and quantify the explanatory variables that influence the four response variables (residence time and particle fate on both the Sacramento and San Joaquin rivers). The second was to use the statistical models to reconstruct residence times and particle fates during periods for which we lacked DSM2 estimations but had hydrologic data from DWR’s Dayflow website. DSM2 only allows particle tracking back to 1989, whereas water exports increased exponentially beginning in 1967 (Fig. S6). To test for a relationship between hydrodynamics and chlorophyll a, we needed the hydrodynamic variables to extend to periods before water exports had reached modern levels and chlorophyll a had declined. Categories of models were fit and compared that had one of four response variables: residence time on the Sacramento River, residence time on the San Joaquin River, particles lost on the Sacramento River, and particles lost on the San Joaquin River. The residence time and particle fate models were fit to data from four of the six water years for which we had DSM2 output: 1991, 1998, 1999, and 2009. These years were selected to include the water years with the lowest and highest combined flow on the Sacramento and San Joaquin rivers (1991 and 1998) and two intermediate years within the available range for DSM2 (1989–2012).

For residence time on the Sacramento and San Joaquin rivers, the models were fit in R using the function lm (R Core Team 2018). Log_10_ transformations were applied to residence time for both rivers to linearize relationships between predictors and responses. For the Sacramento River residence time models, predictors included log_10_ transformed average monthly flow at Freeport (m^3^ s^−1^), the average flow of the next month (m^3^ s^−1^, log_10_ transformed), average monthly CVP plus SWP pumping (m^3^ s^−1^), an interaction between average monthly flow and pumping, agricultural diversions (m^3^ s^−1^), and an interaction between monthly flow and agricultural diversions (Table S1). Predictors for the San Joaquin River residence time models were identical (Table S1), except that monthly flow at Vernalis was used rather than at Freeport (locations in Fig. 1). The monthly averages were calculated from the 15th of each month to the 14th of the following month to match the DSM2 simulations.

For the particle fate models (one set for the Sacramento and one for the San Joaquin), the response variables were the proportion of the 1000 particles that were lost via either agricultural diversions or CVP and SWP exports rather than exiting toward the Pacific Ocean. Beta-binomial models were fit because the response variables were over-dispersed, proportional data (McElreath 2016). Similar suites of predictors were used as for the residence time models described above, with identical transformations and units (Table S2).

The models in each of the four categories were compared with Akaike Information Criterion corrected for small sample size (AIC_c_; Burnham and Anderson 2002). The top-ranked model from each category was then validated against the two water years to which the models were naive (1996 and 2005). Using the predictions from each of the four statistical models for both water years (predictions) and DSM2 derived data (observed), we calculated three model validation metrics: R^2^, Nash-Sutcliffe efficiency, and the Willmott Index of Agreement (Moriasi et al. 2007).

### Reconstruction of Hydrodynamic Variables (Step 4)

Following validation, the statistical models were used to estimate monthly residence times and particle fates (% lost) from Aug 1956 to Oct 2014 based on flow and CVP and SWP pumping data from DWR’s Dayflow website and agricultural diversion data extracted from DSM2 (Fig. 2). For both the Sacramento and San Joaquin rivers, the particles lost (%) variable was converted to particles remaining (%) by subtracting it from 100%. The proportion of particles remaining for each river was then multiplied by the respective river flow to obtain *remaining flow*, or the flow of each river that reaches the Exit in Fig. 1 (i.e., Martinez, CA).

### Chlorophyll a Analysis (Step 5)

Once the monthly hydrodynamic variables were reconstructed, a series of statistical models were built to quantify their influence, and the influence of *P. amurensis*, on chlorophyll a in the SFE (stations D7, D8, and D10; Fig. 1) (Fig. 2). Annual means of chlorophyll a for each station were used as the response variable for two reasons. First, phytoplankton generation times are on the order of days and therefore respond quickly to environmental conditions (e.g., Cloern et al. 1983). Thus, modeling mean annual chlorophyll rather than monthly chlorophyll removed the potential for temporal autocorrelation among the measurements and simplified the analysis. Second, we were interested in drivers of interannual rather than seasonal variability. We had a complete dataset from 1969 to 2014, so sample size was 138 (46 years × 3 stations). Linear models with Gaussian error distributions were fit in which log_10_-transformed mean annual chlorophyll a (µg L^−1^) was the response variable. Predictors included presence/absence of *P. amurensis* (≥1987: present, <1987: absent), Sacramento River residence time (days), flow (m^3^ s^−1^) of Sacramento River water that remains in the SFE (i.e., flows past Martinez, CA), the interaction between residence time and remaining flow for the Sacramento River, the identical variables for the San Joaquin River, and a variable for station (Table 1). We calculated the ‘remaining flow’ variables by multiplying the proportion of particles that were not lost on both rivers by flow in the respective river, either at Fremont (Sacramento River) or Vernalis (San Joaquin River). Each remaining flow variable was log_10_-transformed to linearize its relationship with log_10_-chlorophyll a. The station variable was used to account for potential differences in chlorophyll a among stations. Fourteen models were fit and compared using AIC_c_ (Burnham and Anderson 2002; Table 1).

**Table 1:** Chlorophyll a model comparison

Model	ΔAIC_c_	df	AIC_c_ wt
~Clams + SRT + SFM + SRT × SFM + SJRT + SJFM + SJRT × SJFM	0.0	9	0.8929
~Clams + SRT + SFM + SRT × SFM + SJRT + SJFM + SJRT × SJFM + Station	4.3	11	0.1034
~Clams + SRT + SFM + SRT × SFM + SJRT + SJFM	12.3	8	0.0019
~Clams + SRT + SFM + SRT × SFM + SJRT	12.9	7	0.0014
~Clams + SRT + SFM + SRT × SFM	16.6	6	<0.001
~Clams + SRT + SFM + SRT × SFM + SJFM	18.8	7	<0.001
~Clams + SJRT + SJFM + SJRT × SJFM	20.5	6	<0.001
~Clams + SRT	44	4	<0.001
~Clams + SRT + SFM	45.9	5	<0.001
~Clams + SFM	47.9	4	<0.001
~Clams + SJRT + SJFM	51.2	5	<0.001
~Clams	54.4	3	<0.001
~Intercept	268.4	2	<0.001
~SJRT	270.4	3	<0.001

*Note*: Clams is a dummy variable for the presence/absence of *P. amurensis*, SRT is Sacramento River residence time (days), SFM is Sacramento River volume (m^3^ s^−1^) past Martinez (log_10_-transformed), SJRT is San Joaquin River residence time (days), SJFM is San Joaquin flow past Martinez (log_10_-transformed), and S is station

*ΔAIC*_*c*_ difference between model of interest and top-ranked model in Akaike Information Criterion Units corrected for small sample size

*df* degrees of freedom, *AIC*_*c*_*wt* Akaike weight

*P. amurensis* was included as a predictor because it is well-known to suppress phytoplankton abundance in the SFE (Alpine and Cloern 1992; Thompson 2005; Kimmerer and Thompson 2014). *P. amurensis* was treated as a dummy variable rather than as a density because (1) of the three stations only D7 had density data, (2) benthic invertebrate density data only extended back to 1979, whereas chlorophyll a data extended back to 1969, and (3) other studies have treated *P. amurensis* as ‘present/absent’ because its proliferation was so rapid (e.g., Jassby et al. 2002, although see Kimmerer and Thompson 2014). Residence time was included because it drives phytoplankton productivity (e.g., Soballe and Kimmel 1987; Jassby and Powell 1994 and references therin). Remaining flow was included because we hypothesized that residence time and flow would interact. Our rationale was that chlorophyll a in the SFE should depend on the residence time of the freshwater inputs, as well as the quantity of water experiencing that residence time. While nutrient concentrations drive phytoplankton dynamics in many systems, they were not included as predictors because the data only extend back to 1975 whereas chlorophyll a data extend back to 1969, and because nutrients in the SFE are largely considered replete (e.g., Jassby and Powell 1994).

### Effect Size Calculations

Because DSM2 was explicitly used to quantify the influence of CVP plus SWP pumping on residence time and particle fate, we report the effect size—the difference in the response variable calculated at the minimum and maximum of a predictor variable—of pumping based on the output directly rather than the statistical models of DSM2. In addition, the top-ranked statistical models were used to quantify the influence of each predictor on each of the five response variables (Sacramento and San Joaquin residence times and particle fates, and SFE chlorophyll a). For each predictor, model estimates were made at its minimum and its maximum, with the other variables held constant at their means. For interactions, predictions were made at the minimum and maximum of both factors in the interaction, with the other variables held at their means. However, certain predictions for interactions can be unrealistic. For example, the chlorophyll a model predicts unrealistically high chlorophyll a at maximum remaining flow and maximum residence time, a combination which could not occur (high remaining flows occur during wet years, high residence times occur during droughts; Table 2). Therefore, to calculate environmentally relevant effect sizes, we used the residence time and particle fate models to calculate hydrodynamic conditions during water years back to 1969, but with two different levels of exports (observed and 1977 levels). Then, those hydrodynamic variables were used to make predictions using the chlorophyll a model under four scenarios: 1977 pumping with and without *P. amurensis*, and observed levels of pumping with and without *P. amurensis*. 1977 pumping levels, which were depressed because of a severe drought in CA, were used rather than zero pumping because the lowest level of pumping to which the chlorophyll a model was trained occurred in 1977. The effect sizes are best used to understand the statistical models, including under which conditions certain variables matter most (Table 2), while the estimates during actual water years are better used to interpret the hydrodynamic and biological significance of variables.

**Table 2:** Parameter estimates, 95% confidence intervals (CI), and effect size estimates for the chlorophyll a models (log_10_-transformmed)

Variable	Parameter estimate	95% CI
Intercept	6.933422	4.37, 9.49
Clams	−0.906038	−0.99, −0.82
Sac RT	−0.149171	−0.21, −0.09
Sac Flow M.	−2.350741	−3.39, −1.31
SJ R RT	0.001358	0.00, 0.01
SJ R Flow M.	−0.132087	−0.29, 0.03
Sac RT × Sac Flow M.	0.059996	0.04, 0.08
SJ R RT × SJ R Flow M.	0.010948	0.01, 0.02

Model estimates		
Variable	Min	Max
Clams	16.15	2.00
Sac RT	2.98	0.83
Sac Flow M.	1.88	2.22
SJ R RT	1.63	10.87
SJ R Flow M.	1.07	4.50
Sac RT × Sac Flow M.		
Sac RT	6.40 (min flow)	0.12 (min flow)
Sac RT	0.91 (max flow)	15.87 (max flow)
SJ R RT × SJ R Flow M.		
SJ R RT	0.11 (min flow)	1.41 (min flow)
SJ R RT	1.97 (max flow)	3986.9 (max flow)

*Note*: Model estimates were calculated with other variables held constant at their means except for *Clams*, which was held constant at ‘present’ (i.e., not ‘absent’). SRT is Sacramento River residence time (days), SFM is Sacramento River volume (m^3^ s^−1^) past Martinez (log_10_-transformed), SJRT is San Joaquin River residence time (days), SJFM is San Joaquin flow past Martinez (log_10_-transformed)

## Results

### Community, Nutrient, and Export Dynamics

**Fig. 3:**
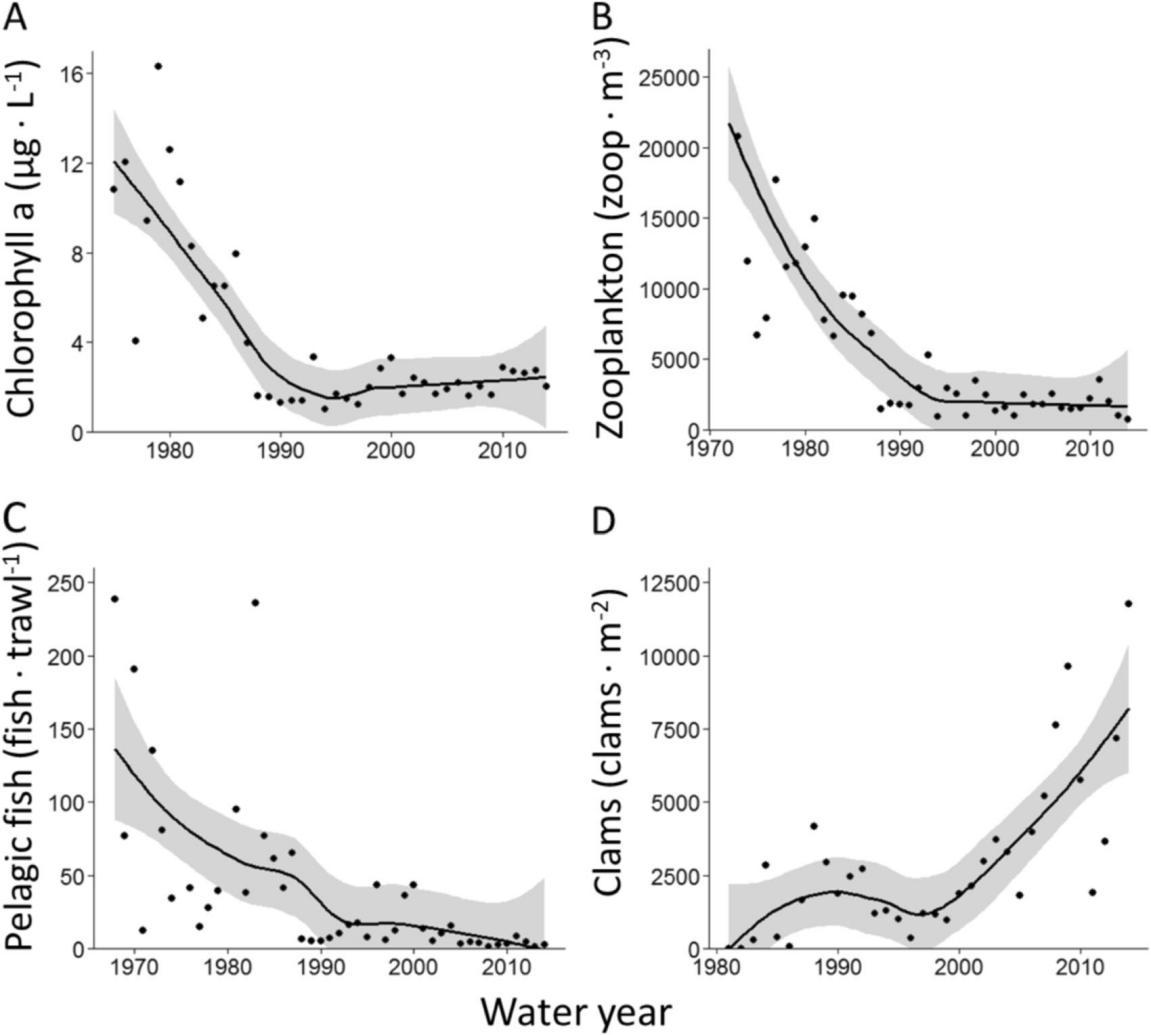
Loess curves fit to water year means of chlorophyll a (panel **a**), mesozooplankton (panel **b**), and pelagic fish (panel **c**) averaged across three stations in the San Francisco Estuary (D10, D8, and D7, or nearby these stations; Table S6). The clam data (panel **d**) are from D7 only (the other stations are not monitored for benthic invertebrates). Note that mesozooplankton, fish, and clams are summed across taxa (although ‘clams’ is 96.4% *P. amurensis*), and that the *x*-axis scales vary

Chlorophyll a, zooplankton, and fish communities of the SFE exhibited steep declines followed by extended periods of low abundance (Fig. 3a–c and S1–S3), while clams increased (mainly *P. amurensis*; Fig. 3d). Mean annual chlorophyll a declined from 11.43 to 2.39 µg L^−1^ between 1975/76 and 2013/14, or 4.8-fold. If the analysis is limited to Aug, Sept, and Oct the chlorophyll a data extends further back: From 1969/70 to 2013/14, chlorophyll a declined from 37.3 to 1.72 µg L^−1^, or 21.7-fold (Aug, Sept, and Oct; Fig. S1D). Zooplankton declined from 27,773 to 876 individuals m^−3^ from 1972/73 to 2013/14, or 31.7-fold. Pelagic fish declined from 157.7 to 1.7 individuals trawl^−1^ from 1968/69 to 2013/14, or 92.3-fold. For chlorophyll a, zooplankton, and pelagic fish, the declines were in progress well before the 1986 invasion of *P. amurensis*, and all exhibited a negative exponential functional form (Fig. 3a–c). Between 1975 and 2014, orthophosphate was fairly stable in the SFE, while dissolved inorganic nitrogen increased (Figs S4 and S5). Mean annual CVP plus SWP exports of fresh water increased exponentially beginning in 1967 (Fig. S6). By ~1990, mean annual exports had somewhat stabilized near 200 m^3^ s^−1^, although with considerable variation depending on flow (Fig. S6).

### DSM2

**Fig. 4:**
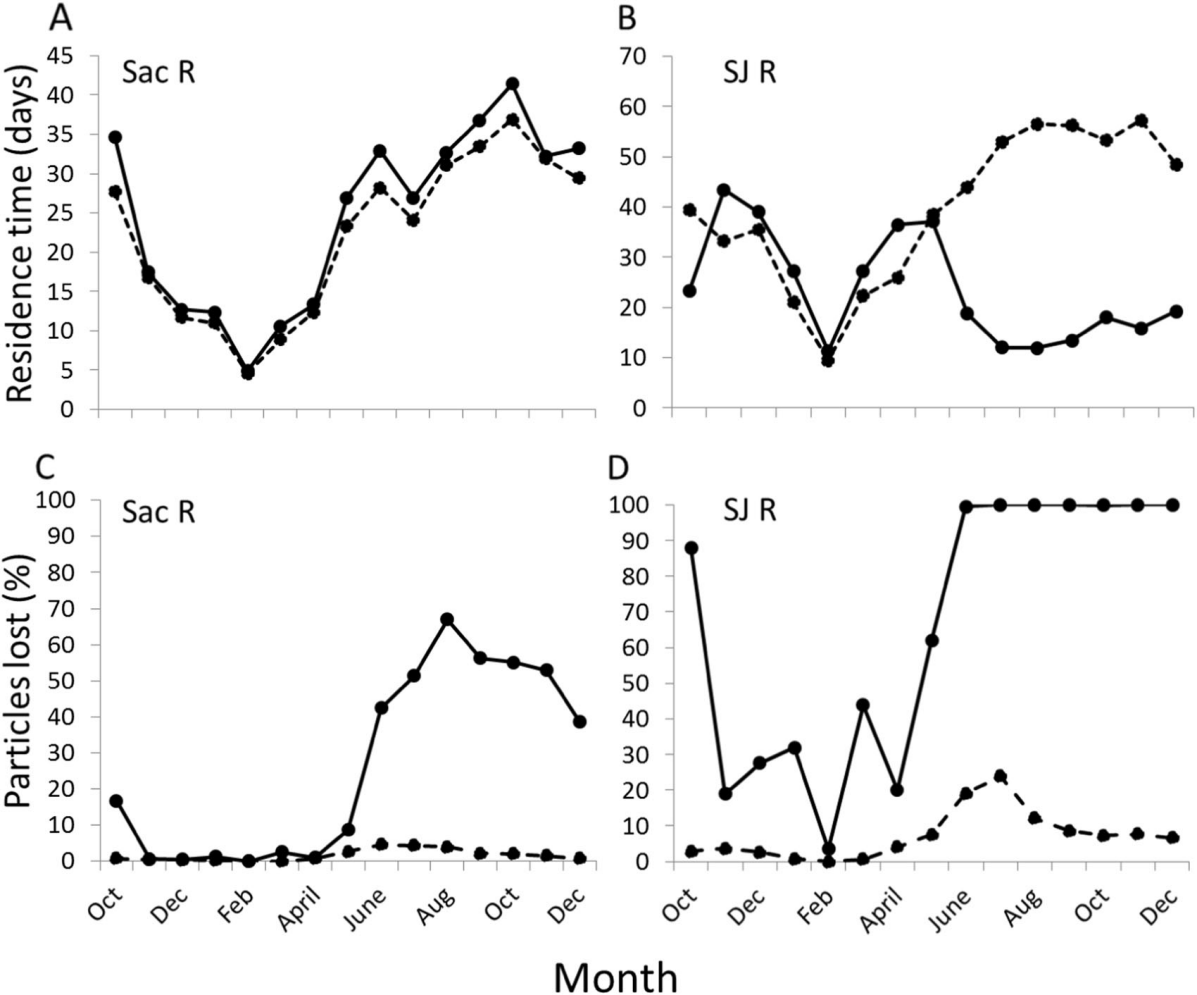
Residence time (**a** and **b**) and particles lost (%; **c** and **d**) with and without observed levels of CVP and SWP pumping for the Sacramento River (**a** and **c**) and Joaquin River (**b** and **d**). Solid lines are ‘CVP and SWP pumping’ and dashed lines are ‘no CVP and SWP pumping’. The plot includes water year 1999 through the first three months of water year 2000 (a period of near average flow)

The top-ranked residence time model for the Sacramento River included flow, agricultural diversions, CVP plus SWP pumping, and an interaction between agricultural diversions and flow (Table S1). Model estimates show that residence time increases with decreasing flow (from 5.1 to 74 days from maximum to minimum flow), with increasing agricultural diversions (from 25.730.3 to 25.730.3 days from minimum to maximum agricultural diversions), and with increasing CVP and SWP pumping (from 25.6 to 31.3 days from minimum to maximum pumping; Fig. 4a). For the San Joaquin River, the top ranked residence time model included flow, CVP and SWP pumping, an interaction between flow and CVP and SWP pumping, agricultural diversions, and an interaction between flow and agricultural diversions (Table S1). Model estimates show that residence time increases with decreasing flow (from 11.8 to 66.8 days), with decreasing agricultural diversions (from 31.2 to 35.5 days), and with decreased CVP and SWP pumping (from 15.5 to 45.8 days; Fig. 4b).

For all three interactions in the residence time models, high flow dampened the influence of the pumps or agricultural diversions on residence time, while low flow increased their influence. For instance, during Aug, Sept, and Oct when flow is low, CVP and SWP pumping exerted a strong, negative effect on residence time on the San Joaquin River (Fig. 4b, S8-S12), and a small, positive influence on residence time on the Sacramento River (Fig. 4a, S8–S12; details in supplement *DSM2: Residence time*). During most years, the negative effect of the pumps on the residence time of the San Joaquin River was diminished during the rest of the year by high flows (Fig. S9–S12). However, during the drought water year of 1991 the pumps exerted a strong, negative influence on residence time nearly year-round (Fig. S8).

The top-ranked particle fate model for the Sacramento River included flow, CVP plus SWP pumping, agricultural diversions, an interaction between flow and agricultural diversions, and an interaction between flow and CVP plus SWP pumping (Table S2). Model estimates show that percent particles lost (to agricultural diversions and CVP plus SWP) decreases with increasing flow (from 34.7 to 0.4% loss) and increases with increasing agricultural diversions (4.2 to 34.4%) and CVP and SWP pumping (2.2 to 72%). The top-ranked particle fate model for the San Joaquin River included flow, CVP plus SWP pumping, agricultural diversions, a flow by CVP plus SWP pumping interaction, and a CVP plus SWP by agricultural diversions interaction (Table S2). Similar to the Sacramento River model, estimates for the San Joaquin River show that percent particles lost (to agricultural diversions and CVP plus SWP pumping) decreases with increasing flow (from 86.1 to 1.7% loss) and increases with increasing agricultural diversions (26.9 to 79.7%) and CVP and SWP pumping (7.0 to 99.6%). Based on DSM2 output, in Aug, Sept, and Oct, when the effects of the CVP and SWP were pronounced (e.g., Fig. 4), the percent of particles lost increased with CVP and SWP pumping from 1.4 to 45.5% on the Sacramento River and 6.0 to 92.1% on the San Joaquin River (details in Supplement *DSM2: Particle fate*).

**Fig. 5:**
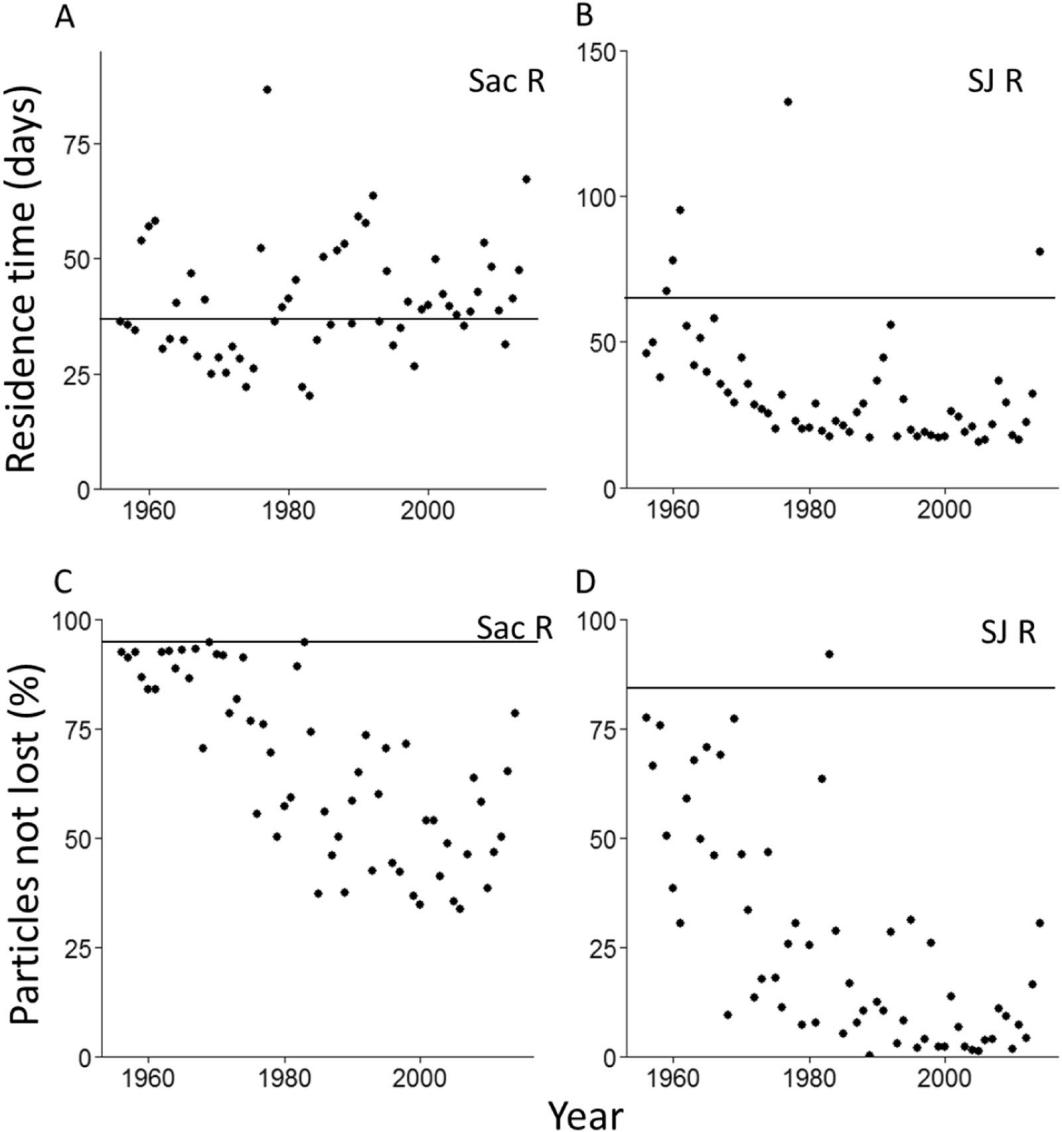
The top two plots are residence time averaged over Aug, Sept, Oct for the Sacramento (panel **a**) and San Joaquin (panel **b**) rivers by calendar year (1956–2014). The lower two plots show the estimated percent of particles that were not lost to agricultural diversions and CVP plus SWP pumping over Aug, Sept, and Oct by calendar year for the Sacramento (panel **c**) and San Joaquin (panel **d**) rivers. All four plots are estimates of actual hydrological conditions (i.e., estimated at actual export rates, flows, and agricultural diversion rates) using our top-ranked hydrodynamic statistical models (Table S1). The horizontal lines on each panel represent mean model predictions with the CVP and SWP pumps set to zero

Each of the four top-ranked models of DSM2 results were successfully cross-validated. The R^2^ of the relationships between DSM2 results and predictions ranged from 0.86 to 0.98 (Table S3). Nash-Sutcliffe efficiencies ranged from 0.85 to 0.97, and Indexes of Agreement ranged from 0.95 to 0.99 (Table S3). Together, these metrics indicate that all four statistical models can be considered ‘very good’ (Moriasi et al. 2007, Figs S13–S16, Table S3). Therefore, the four statistical models were used to reconstruct residence times and particle fates back to 1956, at observed levels of CVP and SWP pumping, with pumping in each statistical model set to zero (Fig. 5), and set to low (1977, a drought year) levels for use in the chlorophyll a models. Predicted residence time for the Sacramento River did not change systematically through time (Fig. 5a). In contrast, San Joaquin River residence time, and particles remaining in both rivers exhibited negative exponential declines (Fig. 5b–d), similar in functional form to the declines in the pelagic community of the SFE (Fig. 3a–c).

### Chlorophyll a

**Fig. 6:**
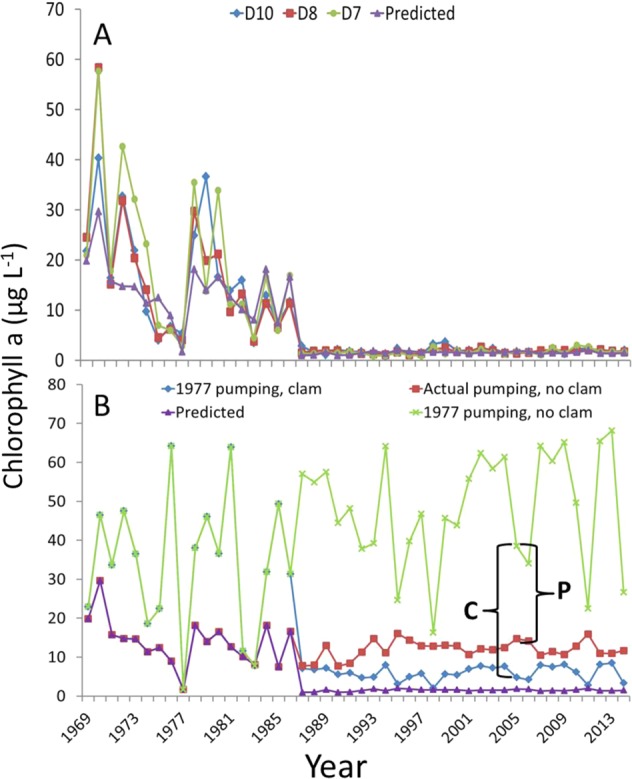
Panel **a** shows chlorophyll a measurements from the SFE, averaged over Aug, Sept, and Oct, at D10, D8, and D7, and model predictions (purple triangles) by calendar year. Panel **b** shows the same model predictions (purple triangles), model predictions at 1977 CVP and SWP pumping with *P. amurensis* (blue diamonds), model predictions at observed levels of exports but no *P. amurensis* (red squares), and 1977 CVP and SWP pumping without clams (green crosses). Quantity C shows the effect size of *P. amurensis* in 2005, quantity P shows the effect size of CVP plus SWP pumping in 2005, using 1977 levels of exports as the minimum (40.7 m^3^ s^−1^), not zero. Note that at high flow (e.g., 1995, 1998, 2006, and 2011), chlorophyll a is depressed by low residence time, reducing the negative influence of exports and *P. amurensis*

The top-ranked chlorophyll a model included the *P. amurensis* dummy variable, San Joaquin River residence time, remaining San Joaquin River flow, an interaction between San Joaquin residence time and remaining San Joaquin River flow, Sacramento River residence time, remaining flow of the Sacramento River, and an interaction between Sacramento residence time and remaining Sacramento River flow (Table 1). Chlorophyll a predictions from the model agreed well with actual measurements, with an R^2^ of 0.78 between observed and predicted chlorophyll a (Fig. 6a). The presence of *P. amurensis* strongly suppressed chlorophyll a (Table 2). While chlorophyll a strongly increased with residence time when remaining San Joaquin River flow was high, at low remaining flow there was little influence of residence time on chlorophyll a (Table 2). A similar pattern occurred on the Sacramento River, where the increasing residence time had a positive influence on chlorophyll a, but only when the remaining flow was substantial (Table 2). Thus, chlorophyll a in the SFE is strongly driven by *P. amurensis* and the interaction between residence time and remaining freshwater flow. Because CVP and SWP exports reduce residence time on the San Joaquin River, reduce remaining flow of the San Joaquin River, reduce remaining flow of the Sacramento River, and exerts only a slightly positive influence on Sacramento River residence time, CVP and SWP exports had an overall negative influence on chlorophyll a (Fig. 5).

With exports at 1977 levels and without *P. amurensis*, estimated chlorophyll a was 47.3 µg L^−1^ averaged over 1990–2014 (after *P. amurensis* was established and exports had reached modern levels, Fig. S6). At 1977 levels of CVP and SWP exports but with *P. amurensis*, model estimated chlorophyll a declined from 87.6% to 5.9 µg L^−1^ (Fig. 6b). At observed export levels but without *P. amurensis*, model estimated chlorophyll a was 12.2 µg L^−1^, a 74.1% decline (Fig. 6b). At observed export levels and with *P. amurensis*, estimated chlorophyll was 1.5 µg L^−1^, a 96.8% decline (Fig. 6b). Thus, we estimate that the SFE lost an average of 45.8 µg L^−1^ of chlorophyll a during Aug, Sept, and Oct due to the combined influence of freshwater exports and *P. amurensis* averaged over 1990–2014 (Fig. 6b). We note that the 1977 level of exports was used rather than zero as the minimum level of freshwater exports, so the influence of CVP and SWP pumping on chlorophyll a is likely larger than our estimates (i.e., 1977 CVP plus SWP exports were 40.7 m^3^ s^−1^, not zero).

The effect sizes of CVP and SWP exports and *P. amurensis* depended on water year type. In the presence of *P. amurensis* and excluding the four years with the highest Aug, Sept, Oct flow in the Sacramento and San Joaquin rivers (1995, 1998, 2006, and 2011), the model estimated loss of chlorophyll a due to CVP and SWP pumping was 39.9 µg L^−1^ (declining from 51.7 to 11.8 µg L^−1^). During the same period, the estimated loss due to *P. amurensis* was 45.3 µg L^−1^, declining from 51.7 to 6.4 µg L^−1^. In contrast, during the four wet years, the model estimated loss due to exports was just 9.6 µg L^−1^, declining from 24.4 to 14.7 µg L^−1^. During the four wet years the estimated loss for *P. amurensis* was 21.3 µg L^−1^, declining from 24.4 to 3.02 µg L^−1^. Together, CVP and SWP exports and *P. amurensis* reduced model estimated mean chlorophyll a from 51.7 to 1.5 µg L^−1^ during the drier periods, and from 24.4 to 1.8 µg L^−1^ during the four wet years.

## Discussion

There is a growing consensus that the decline in pelagic fish abundance in the SFE is at least partially due to a trophic cascade, triggered by declining phytoplankton (Feyrer et al. 2003; Sommer et al. 2007; Hammock et al. 2017; Hamilton and Murphy 2018). Whereas a top-down trophic cascade would be expected to cause decreases in only certain trophic levels (e.g., fish and phytoplankton, Brett and Goldman 1996), the observational data we present is more consistent with a bottom-up trophic cascade. That is, the SFE exhibited similarly timed, negative exponential declines in phytoplankton, zooplankton, and pelagic fish abundance (Fig. 3a–c). While the invasion of *P. amurensis* is a well-established cause for the phytoplankton decline (Alpine and Cloern 1992; Thompson 2005), others have suggested that CVP and SWP exports may also reduce phytoplankton abundance, potentially by exporting phytoplankton and reducing residence time (Jassby and Powell 1994; Arthur et al. 1996; Jassby et al. 2002). Here, we use a series of modeling steps to determine the influence of CVP and SWP exports on phytoplankton, while accounting for the impacts of grazing caused by the invasion of *P. amurensis* (Alpine and Cloern 1992). Model estimates indicate that CVP and SWP water exports and *P. amurensis* each exert a potent, negative influence on phytoplankton in the SFE, supporting the hypotheses of both Alpine and Cloern (1992) and Jassby and Powell (1994).

The interactions between residence time and remaining flow on each river are key to understanding how freshwater exports from the south Delta reduce phytoplankton tens of kilometers away from the SFE (Fig. 1). Residence time has long been known to be an important driver of phytoplankton abundance in aquatic ecosystems, with decreased residence time associated with lower concentrations of chlorophyll a (e.g., Soballe and Kimmel 1987). However, the associations Søballe and Kimmel (1987) described are for the waterbodies themselves (e.g., lakes and rivers). In the SFE, the Sacramento and San Joaquin rivers mix with water from the Pacific Ocean, so the volume of fresh water on which residence time acts is also important. This is best illustrated by extreme water years. During the drought year of 1977, residence times on the Sacramento and San Joaquin peaked (Figs. 5a, b), but SFE phytoplankton was atypically low (Fig. 6a). According to the top-ranked chlorophyll a model, phytoplankton did not respond to elevated residence time because remaining flow was low. That is, there was insufficient fresh water experiencing long residence times to support abundant phytoplankton in the SFE (although see Nichols (1985) and Cloern et al. (1983) for alternative explanations). The opposite occurred during 1983, an extremely wet year. While remaining freshwater flow was high, chlorophyll a in the SFE was suppressed because residence times on both rivers were low (Figs. 5 and 6). During 1970, in contrast, *P. amurensis* had not yet invaded, residence times were moderate, and CVP and SWP exports were relatively low (Figs. 5, 6, S17, and S18). This left a high proportion of inflow to experience the moderate residence times, resulting in abundant phytoplankton (Fig. 6a).

Model estimates indicate that pumping by the CVP and SWP exerts a potent, negative influence on SFE phytoplankton during periods of moderate flow. However, we note that the true effect size of CVP and SWP could not be estimated because the minimum export rate during summer/fall over the study period (1969–2014) was over 40 m^3^ s^−1^, making our effect size estimates of CVP and SWP exports on chlorophyll a conservative. According to model estimates, if summer/fall pumping had been reduced to 1977 levels from 1990 to 2014, in the presence of *P. amurensis*, mean chlorophyll a would have increased from 1.5 to 5.9 µg L^−1^. For reference, growth of *Daphnia magna* is maximized at approximately 10 µg L^−1^ of chlorophyll a in freshwater portions of the SFE (Müller-Solger et al. 2002). The negative effect on chlorophyll a caused by CVP plus SWP pumping was larger in moderate water years and smaller in wet years. We stress, however, that the estimates we report should not be considered an indication of how the SFE would respond to reduced summer/fall exports in the future because the influence of *P. amurensis* on chlorophyll a could change unpredictably with reduced summer/fall exports. The chlorophyll a model was not trained to periods of low exports and moderate flow following the invasion of *P. amurensis*, because this combination of conditions never occurred. Populations of *P. amurensis* and other grazers may increase in response to elevated phytoplankton (e.g., Beukema and Cadée 1986), potentially muting the response of phytoplankton to the more favorable hydrodynamics. Alternatively, the additional freshwater flow would move the salinity field seaward, potentially shifting the range of *P. amurensis* down-estuary and reducing the influence of the bivalve (Peterson and Vayssieres 2010). The introduction of *Microcystis* to the ecosystem, first reported in 1999 (Lehman and Waller 2003, Lehman and Lehman et al. 2005), adds to the uncertainty because any management actions that improve hydrodynamic conditions for phytoplankton are likely to also promote *Microcystis* in the Delta. Thus, given the novel SFE/Delta community, a substantial decrease in summer/fall exports could conceivably lead to a variety of outcomes, including: little change in phytoplankton and more abundant clams, harmful algal blooms in the Delta, or higher phytoplankton densities. These ecological uncertainties suggest that an adaptive management approach to summer/fall pumping rates, in combination with foodweb monitoring, is warranted.

One important outcome of our study is that the reduction of chlorophyll a by the CVP and SWP pumps is most profound at lower river flows. During periods of high flow, residence times and remaining flow are relatively unaffected by CVP and SWP pumping (e.g., spring and early summer in Fig. 4), so the exports exert little influence on chlorophyll a (e.g., 1998 in Fig. 6b). During these high-flow periods, chlorophyll a is low largely because of low residence times, and less due to the influence of *P. amurensis* and exports (Fig. 6b, Fig. S10). At lower flows in summer and fall, or nearly year-round during droughts (Fig. S8), the influence of the CVP and SWP pumps on SFE hydrodynamics—and therefore phytoplankton—increases. For example, based on our DSM2 results, the loss of residence time due to exports on the San Joaquin River in April, May, and June was 10.2 days, while during Aug, Sept, and Oct it was 36.9 days. This magnitude of residence time loss is substantial, given that phytoplankton abundance can double within a few days (e.g., 2.9 day doubling time along shoals in the SFE, Cloern et al. 1983). We note that the suppression of phytoplankton abundance due to exports cannot be reversed with equivalent releases from upstream reservoirs. Releasing water in late summer/fall increases flow, which decreases residence time, and therefore suppresses phytoplankton abundance (Table 2, Fig. 6).

Most estuaries with strong human influence exhibit elevated productivity due to nutrient inputs (e.g., Schulz et al. 1991; Kemp et al. 2005), and on a global scale there is a strong correlation between total nitrogen inputs and phytoplankton production (Anderson et al. 2002). In the SFE, several studies have concluded that nitrogen itself suppresses phytoplankton productivity. For example, several authors suggest that high ammonia depresses phytoplankton production in the SFE by reducing nitrogen uptake (Dugdale et al. 2007; Parker et al. 2012; Wilkerson and Dugdale 2016). Glibert et al. (2011) concluded that nitrogen should be reduced to restore the foodweb. Others have concluded that nutrients are replete in the SFE, and point to factors such as flow and grazing to explain interannual variation in chlorophyll (e.g., Jassby and Powell 1994; Jassby et al. 2002; Dahm et al. 2016). Given that hydrodynamics and *P. amurensis* together explained 78% of the interannual variation in chlorophyll a in our study, we conclude that SFE phytoplankton productivity is low in spite of high nutrients, not because of them.

While our chlorophyll a model performed well, we acknowledge that it could be improved. Although we included an interaction between remaining flow and residence time of freshwater inputs, the model might make better predictions if this interaction is refined. Possibly residence time of only the remaining flow should be used, rather than residence time of the Sacramento and San Joaquin river inputs. However, this is uncertain given that some of the water that was exported may contribute to pelagic production if it enters the estuary before being pushed upstream by the tide and exported. Future analyses may also improve on our work by including nutrients—which may interact with hydrodynamics—and biomass of *P. amurensis*.

In conclusion, the SFE has transitioned from a productive estuary with abundant pelagic fish into an unproductive estuary with few pelagic fish, and our analysis suggests that the invasion by *P. amurensis* and CVP and SWP exports are largely responsible. CVP and SWP exports suppress pelagic productivity by reducing residence time on the San Joaquin River, and reducing the freshwater flow on which residence time acts on both the Sacramento and San Joaquin rivers. Had CVP plus SWP pumping remained at 1977 levels from 1990–2014, we estimate that chlorophyll a would have averaged 5.9 rather than 1.5 µg L^−1^ (assuming no change in grazing rates). During high flow periods, the pumps have little influence on the hydrodynamics of the SFE, and therefore exert relatively little influence on pelagic productivity. The opposite is true at low flows. Because there has never been a summer/fall with moderate flow, low exports, but with *P. amurensis*, it is uncertain how the SFE would respond to future reductions in summer/fall exports. These results not only provide some clarity on the causes of the collapse of the SFE pelagic foodweb, but also present an approach that incorporates modeling of hydrodynamic factors with long-term monitoring data to disentangle complex management problems associated with large estuarine ecosystems. Further, the results demonstrate that large-scale freshwater exports from upstream of an estuary can be a potent suppressor of productivity, a result that may be applicable to other heavily managed estuaries.

## Supplementary information

Supplementary material
