# Supplementary material for: Hydrodynamic Modeling Coupled with Long-term Field Data Provide Evidence for Suppression of Phytoplankton by Invasive Clams and Freshwater Exports in the San Francisco Estuary
Source: Environ Manage. 2019 Apr 3;63(6):703–17. doi: 10.1007/s00267-019-01159-6 (PMC6525664; doi:10.1007/s00267-019-01159-6)
Supplement: Supplementary file 1 — Supplementary material [file 267_2019_1159_MOESM1_ESM.docx]

Supplementary material

*Characterizing decadal trends*

Water years in California begin on Oct 1 and end on Sept 30. We calculated water year means for six variables at three locations in the SFE to characterize decadal trends (D7, D8, and D10, Fig. 1). Water year means were calculated at each of the three locations for chlorophyll a, zooplankton abundance, clam abundance, pelagic fish abundance, orthophosphate, and dissolved inorganic nitrogen. By using water year means, we coerced disparate datasets onto the same (water year) scale and eliminated seasonal variance, allowing us to more easily identify trends (Smith 2006). For the calculations of water year means, we used publicly available data on water exports, water quality, and abundance of zooplankton, clams, and pelagic fish. The data were obtained from four sources. Water export and outflow data came from DWR’s Dayflow website, water quality, meso-zooplankton abundance, and clam abundance data were obtained from DWR’s Environmental Monitoring Program. Fish abundance was based on California Department of Fish and Wildlife’s (CDFW) Fall Midwater Trawl surveys, and was downloaded from CDFW’s FTP site: <ftp://ftp.dfg.ca.gov/TownetFallMidwaterTrawl/FMWT%20Data/>. In addition, a US Bureau of Reclamation dataset of chlorophyll a measurements beginning in 1968, the precursor to DWR’s discrete water quality monitoring, was obtained from Alan Jassby (Jassby and Powell 1994). The three locations were selected because (1) they had continuous chlorophyll a measurements (1969 to present), (2) had largely complete community and water quality data, (3) were all within the range of *P. amurensis* (>2 PSU; Nicolini and Penry 2000), and (4) were below the confluence of the Sacramento and San Joaquin rivers, so we expected them to respond to the hydrodynamics of the Sacramento and San Joaquin rivers similarly. The latter point allowed us to analyze the three stations together in terms of the influence of hydrodynamics and *P. amurensis* on chlorophyll a in the final step of our analysis.

Each of the six datasets have their own characteristics and spatial and temporal sampling scales. The Dayflow database provides both daily flow at various points in the SFE and CVP and SWP export rates beginning in 1955. We used Dayflow data on flow of the Sacramento River (at Freeport, USGS Station #11447650), flow of the San Joaquin River (at Vernalis, USGS Station #11303500), and CVP and SWP pumping rates, which we added together and treated as a single variable. The discrete water quality data from DWR begins in 1975, and was collected either monthly or bi-monthly. For the final chlorophyll a analysis, a combination of DWR and Bureau of Reclamation data from Aug, Sept, Oct chlorophyll a data were used (Bureau of Reclamation from 1969-1974 and DWR from 1975-2014). We note that Bureau of Reclamation collected chlorophyll a data from 1968 as well, but all three months were unavailable, so 1968 was not included. The zooplankton data were collected by California Department of Fish and Wildlife (CDFW), beginning in 1972. Most years CDFW sampled zooplankton nine months of the year (Mar-Nov), but occasionally Dec, Jan, and Feb were sampled as well. In addition, samples were collected twice per month from 1972-93, and once per month beginning in 1994. Benthic organism samples were collected monthly by DWR beginning in 1980 at one of the three stations we examined (D7/D7-C). Finally, Fall Midwater Trawl quantifies pelagic fish abundance monthly from Sept-Dec, with the first complete water year of data beginning in 1968. The survey did not occur in 1974 and 1979 so water years 1975 and 1980 were left out of our analysis.

In order to make comparisons across sites and through time long-term monitoring data were standardized as follows: Water quality samples that were collected bimonthly were averaged before calculating water year means, which included total inorganic nitrogen (mg L^-1^), orthophosphate (mg L^-1^), and chlorophyll a (µg L^-1^). We averaged the bi-monthly zooplankton samples for 1972-93 and excluded the winter samples (Dec, Jan, and Feb). Zooplankton counts were summed across taxa to create a ‘zooplankton density’ metric and water year means were calculated as in Hammock et al. (2017). We extracted *C. fluminea* and *P. amurensis* densities from the benthic invertebrate data, summed them, and calculated a combined water year mean for stations D7 and D7-C (although we summed across both taxa, 96.4% of the clams were *P. amurensis*). For the Fall Midwater Trawl data, we summed across all fish taxa to obtain a metric of pelagic fish abundance for each location (individuals trawl^-1^). Of the 40 fish taxa observed at the three stations examined, the five most abundant species were Longfin Smelt (*Spirinchus thaleichthys*), Striped Bass (*Morone saxatilis*), American Shad (*Alosa sapidissima*), Northern Anchovy (*Engraulis mordax***)**, and Delta Smelt (*Hypomesus transpacificus*; from most to least abundant). Loess curves were fit to water year means using R to visualize trends (R core team 2016). The ‘span’ argument in ggplot2, which controls the influence that individual points exert on Loess curves, was set at 0.75 for all variables (Wickham 2016). In addition, means for the first two years and last two years of data for chlorophyll a, zooplankton, and pelagic fish were calculated so that the change in the pelagic foodweb could be quantified. Relationships among sites, locations, and original station names are in Table S6.

*DSM2*

DSM2 was originally calibrated and validated in 1997 (DWR 1997), recalibrated in 2000 (DSM2PWT 2001), recalibrated in 2009 (DWR 2009) and recalibrated again most recently in 2012 (Liu 2012) and 2013 (DWR 2013). The V8.1 update included adjustments to HYDRO’s Manning’s coefficients to match observed flows, an update of the channel cross sections, and an improved dispersion formulation. The model was calibrated from 10/1/2001 to 10/1/2002 and 10/1/2007 to 10/1/2008 and validated from 10/1/2006 to 10/1/2007 and 10/1/2008 to 10/1/2009.

*DSM2: Residence time*

For the Sacramento River, residence time was slightly increased by pumping when 10-50% of particles remained in the estuary, pumping had no influence on residence time when 60% of particles remained, and residence time was slightly decreased by pumping when 70-90% of the particles remained in the estuary (Fig. S7). For the San Joaquin River, particles exited more quickly with CVP and SWP pumping across all levels of remaining particles (Fig. S7).

Based on DSM2 results, during Aug, Sept, and Oct when flows were relatively low, the CVP and SWP pumps reduced residence time by 36.9 days, from 61.2 to 24.4 days on the San Joaquin, and increased residence time on the Sacramento River by 3 days, from 39.0 to 42.0 days (e.g., Figs. 4A, 4B). However, when flows were relatively high during April, May, and June, CVP and SWP exports reduced residence time by only 10.2 days on the San Joaquin River, from 38.5 to 28.3 days (e.g., Fig. 4B), and increased residence time by 1.2 days, from 27.1 to 28.3 days on the Sacramento (e.g., Fig. 4B). Model estimated effect sizes, parameter estimates, and confidence intervals are in Table S4.

*DSM2: Particle fate*

Based on DSM2 output, 28.9 % of Sacramento River particles were lost with the pumps on, and 1.9 % with the pumps off (i.e., lost to agricultural diversions). On the San Joaquin, 71.0 % of particles were lost with the pumps on, and 8.2 % with the pumps off (i.e., lost to agricultural diversions). Based on model estimates, 0.4 % of Sacramento particles were lost at maximum flow, while 34.7 % were lost at minimum flow. On the San Joaquin, 1.4 % of particles were lost at maximum flow, while 83.2 % of particles were lost at minimum flow. The top-ranked models for both the Sacramento and San Joaquin rivers included flow by CVP plus SWP pumping interactions. The Sacramento River model also included an interaction between agricultural diversions and pumping, and the San Joaquin model included an interaction between agricultural diversions and CVP plus SWP pumping (Tables S5).


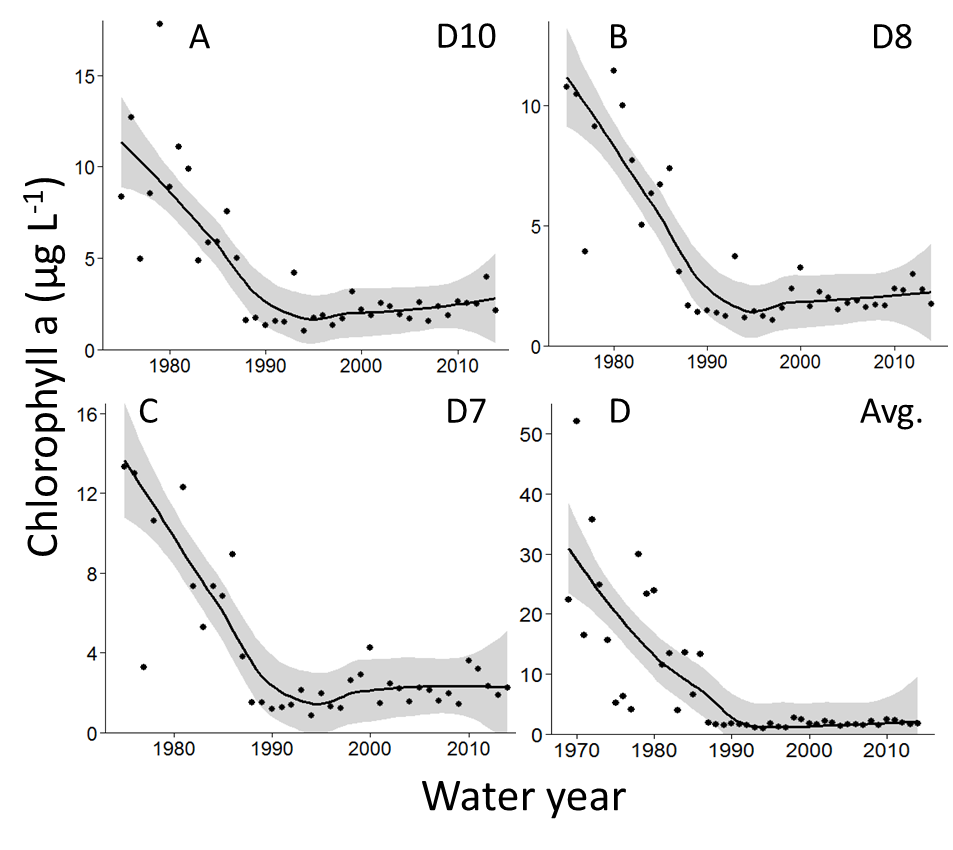


Fig. S1 Panels A-C are mean annual chlorophyll a concentration by water year at three locations in the SFE (Fig. 1, Table S6). Panel D shows the average chlorophyll a concentration at D7, D8, and D10 averaged across Aug, Sept, and Oct, months for which the chlorophyll data extend further back in time. Loess curves are fit to the water year means and the shaded regions represent the 95% confidence intervals. Note that x and y axes vary in scale among the panels.


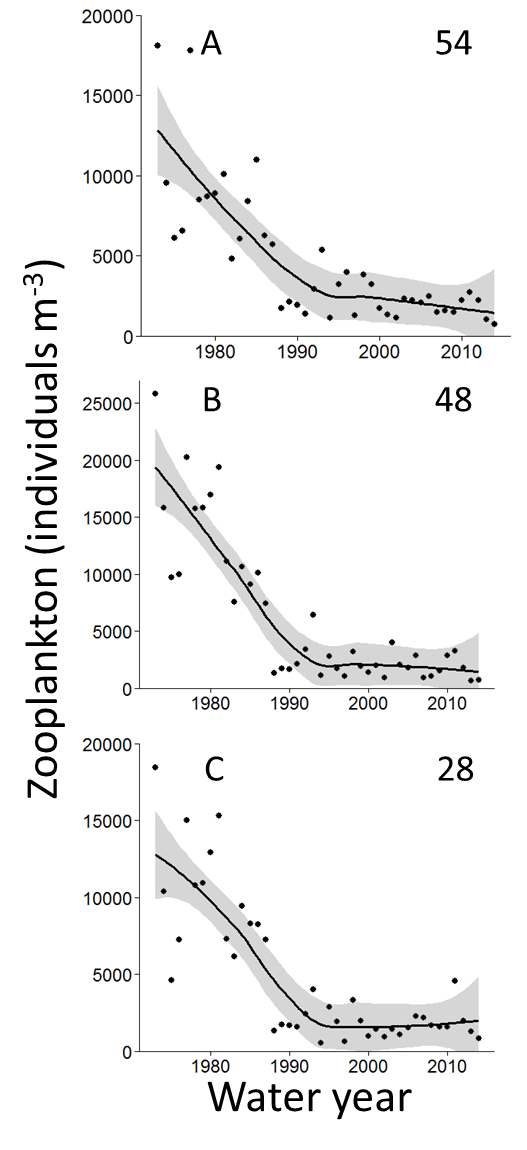


Fig. S2 Mean annual mesozooplankton (individuals m^-3^) by water year at three locations in the SFE (Fig. 1, Table S6). Mesozooplankton were summed across taxa. Loess curves are fit to the water year means and the shaded regions represent the 95% confidence intervals.


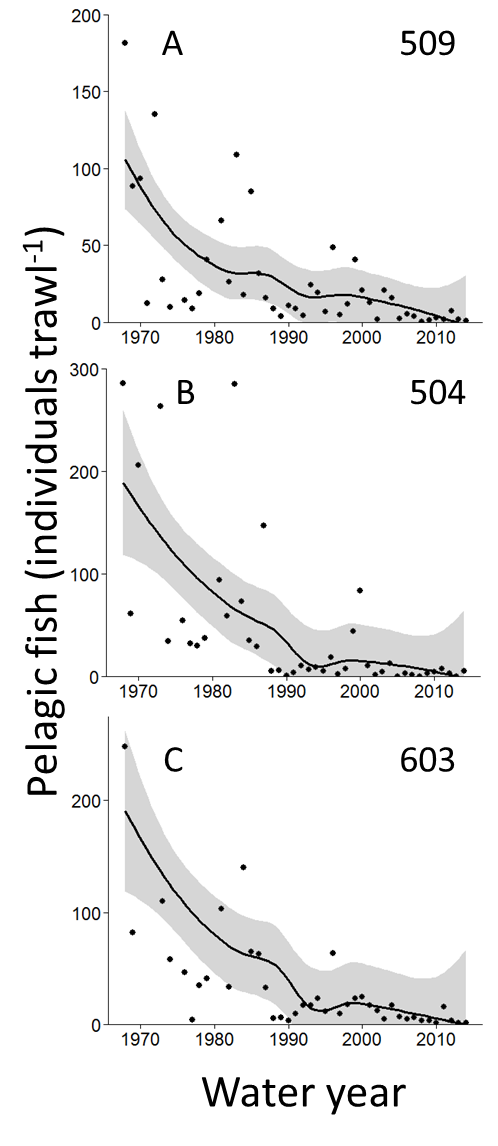


Fig. S3 Mean annual pelagic fish (individuals trawl^-1^) by water year at three locations in the SFE (Fig. 1, Table S6). Fish abundances were summed across taxa. Loess curves are fit to the water year means and the shaded regions represent the 95% confidence intervals.


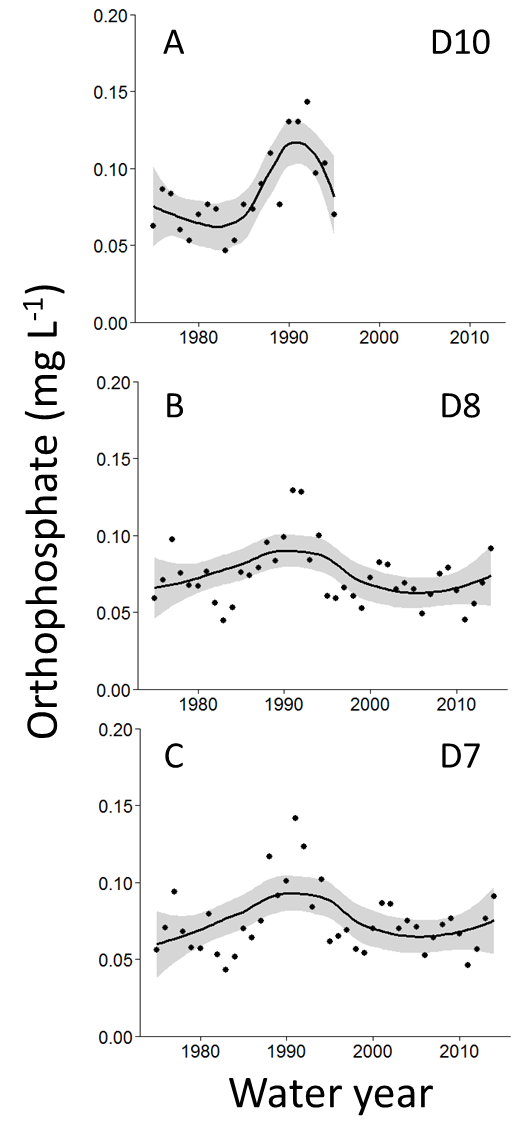


Fig. S4 Mean orthophosphate (mg L^-1^) by water year at three locations in the SFE (Fig. 1, Table S6). Note that water quality data after water year 1995 were not collected at station D10.


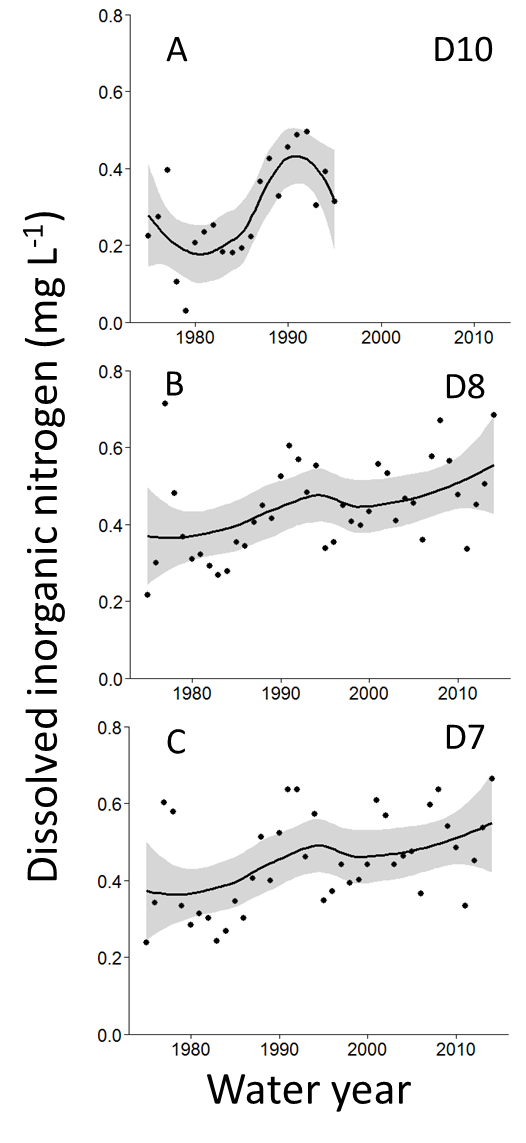


Fig. S5 Mean dissolved inorganic nitrogen (mg L^-1^) by water year at three locations in the SFE (Fig. 1, Table S6). Note that water quality data after water year 1995 were not collected at station D10.


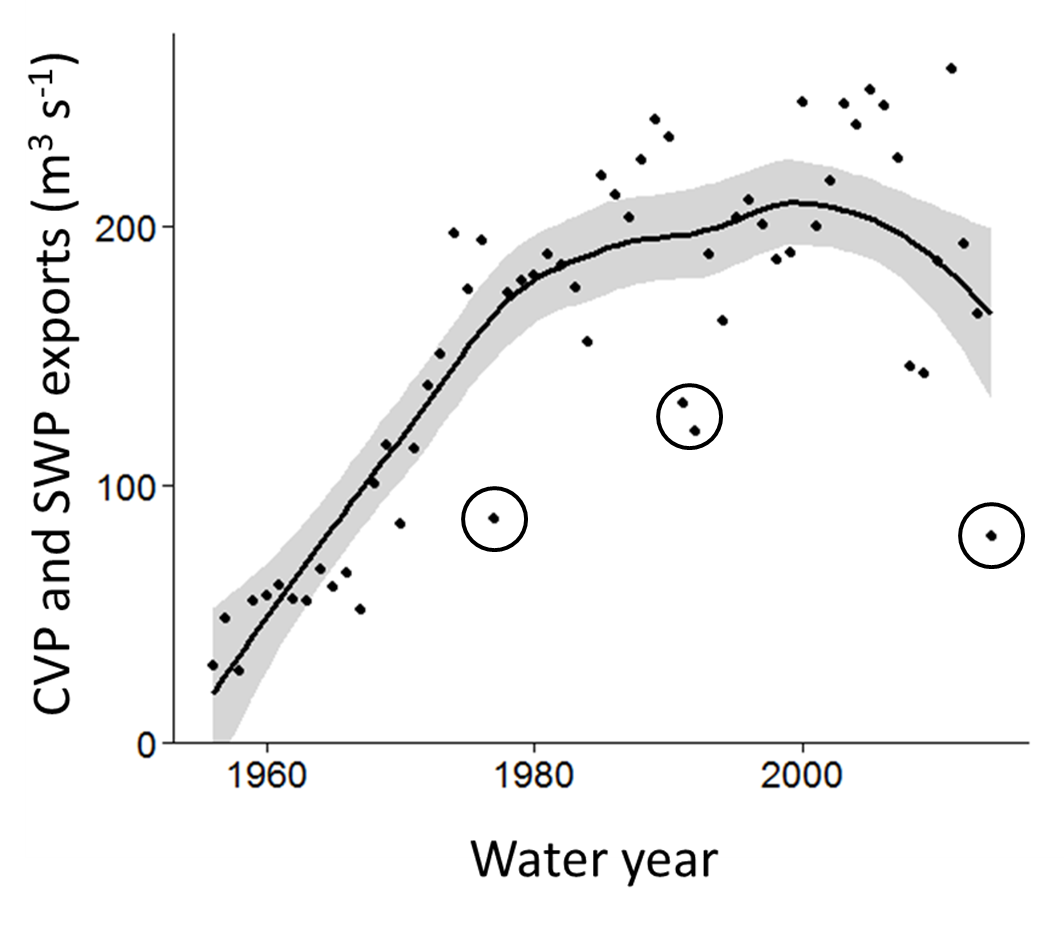


Fig. S6 CVP and SWP water exports (m^3^ s^-1^) by water year. The circled points are water years characterized by drought in California (i.e., 1977, 1991-92, and 2014).


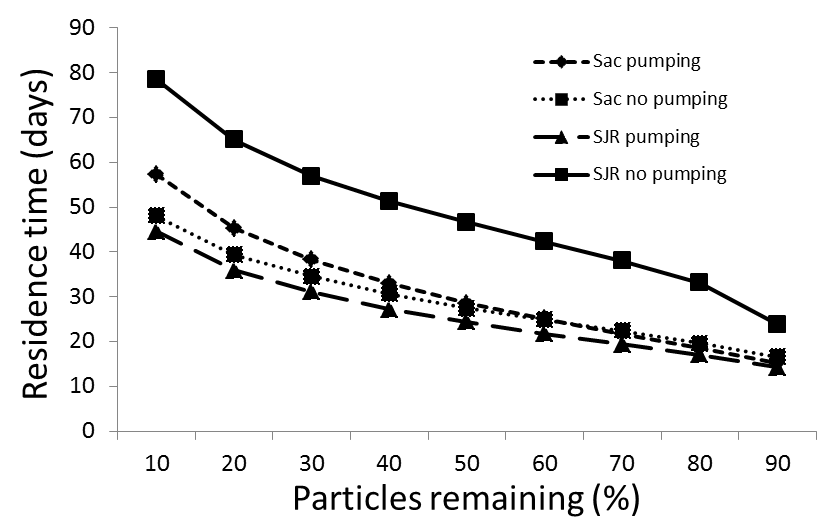


Fig. S7 Residence time (days) as a function of particles remaining (%) in the SFE for the Sacramento and San Joaquin rivers, with and without CVP and SWP pumping. This figure is averaged across all DSM2 simulations (1991, 1996, 1998, 1999, 2005, and 2009 water years plus the three months following each water year).


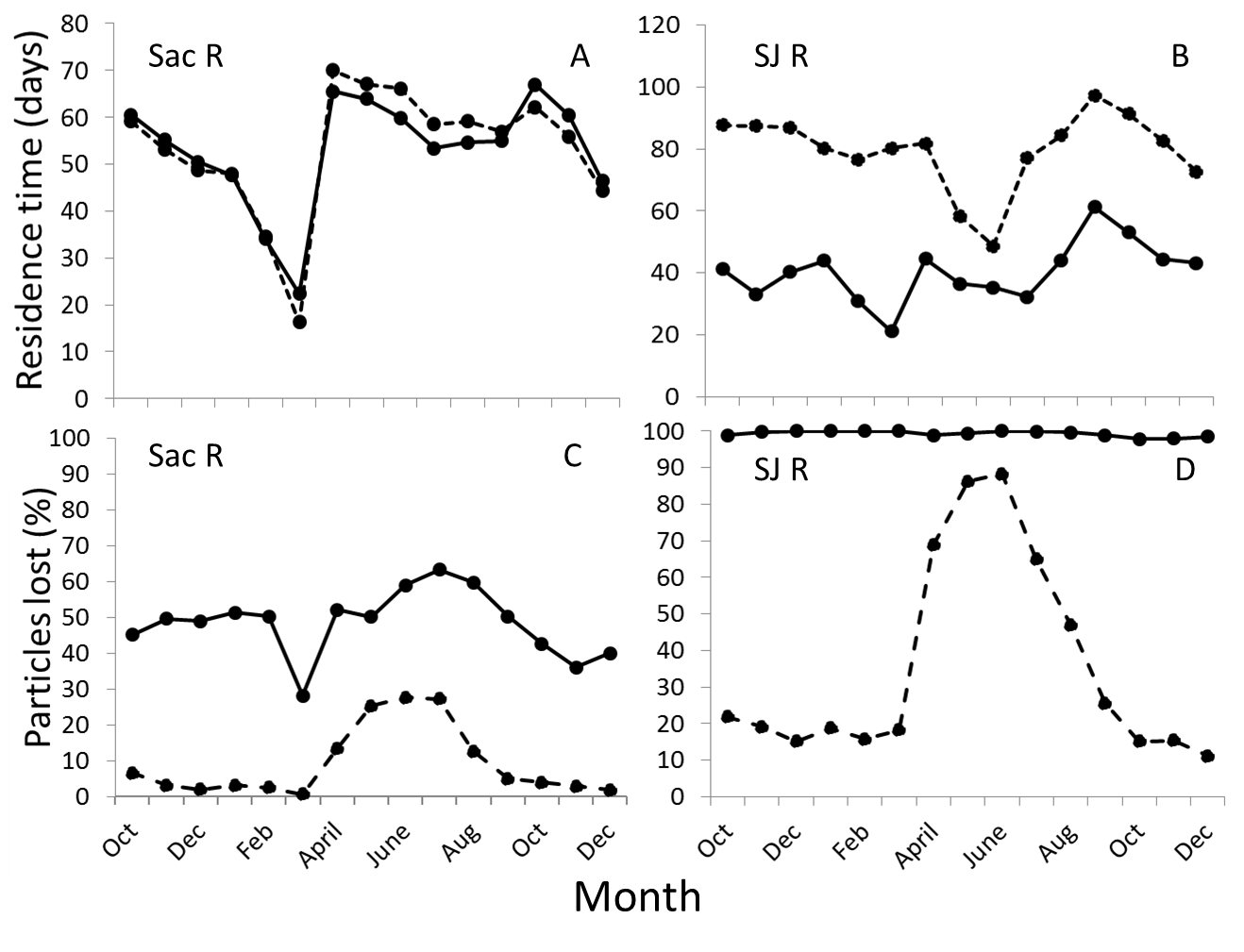


Fig. S8 Residence time (A and B) and percent particles lost (C and D) with and without historical levels of CVP and SWP pumping for the Sacramento River (A and C) and Joaquin River (B and D). Solid lines are ‘pumping’ and dashed lines are ‘no pumping’. The plot includes water year 1991 (a period of extremely low outflow) through the first three months of water year 1992. Particles were released on the 15^th^ of each month.


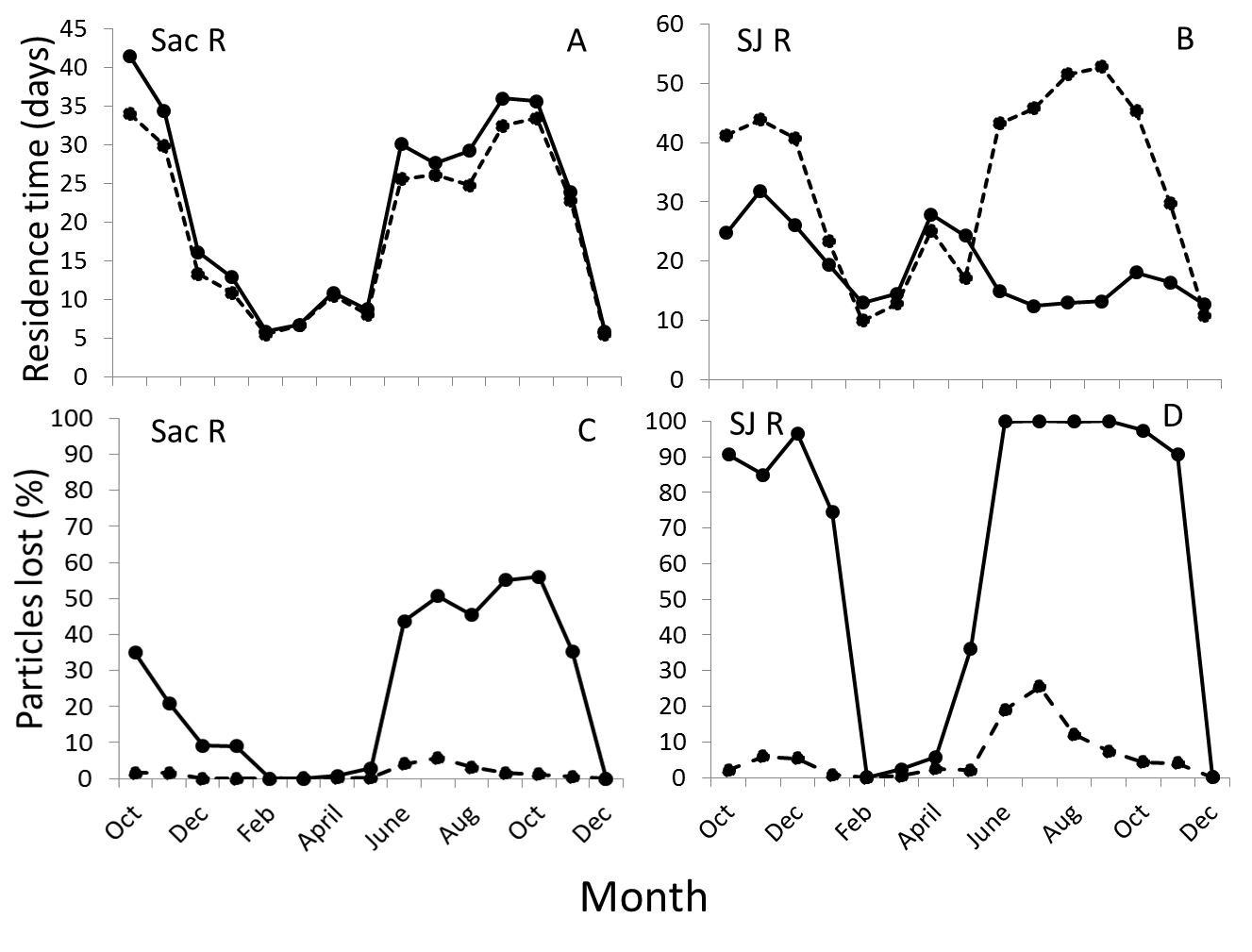


Fig. S9 Residence time (panels A and B) and particles lost (%; panels C and D) with and without historical levels of CVP and SWP pumping for the Sacramento River (panels A and C) and Joaquin River (panels B and D). Solid lines are ‘pumping’ and dashed lines are ‘no pumping’. The plot includes water year 1996 (a period of above average outflow) through the first three months of water year 1997. Particles were released on the 15^th^ of each month.


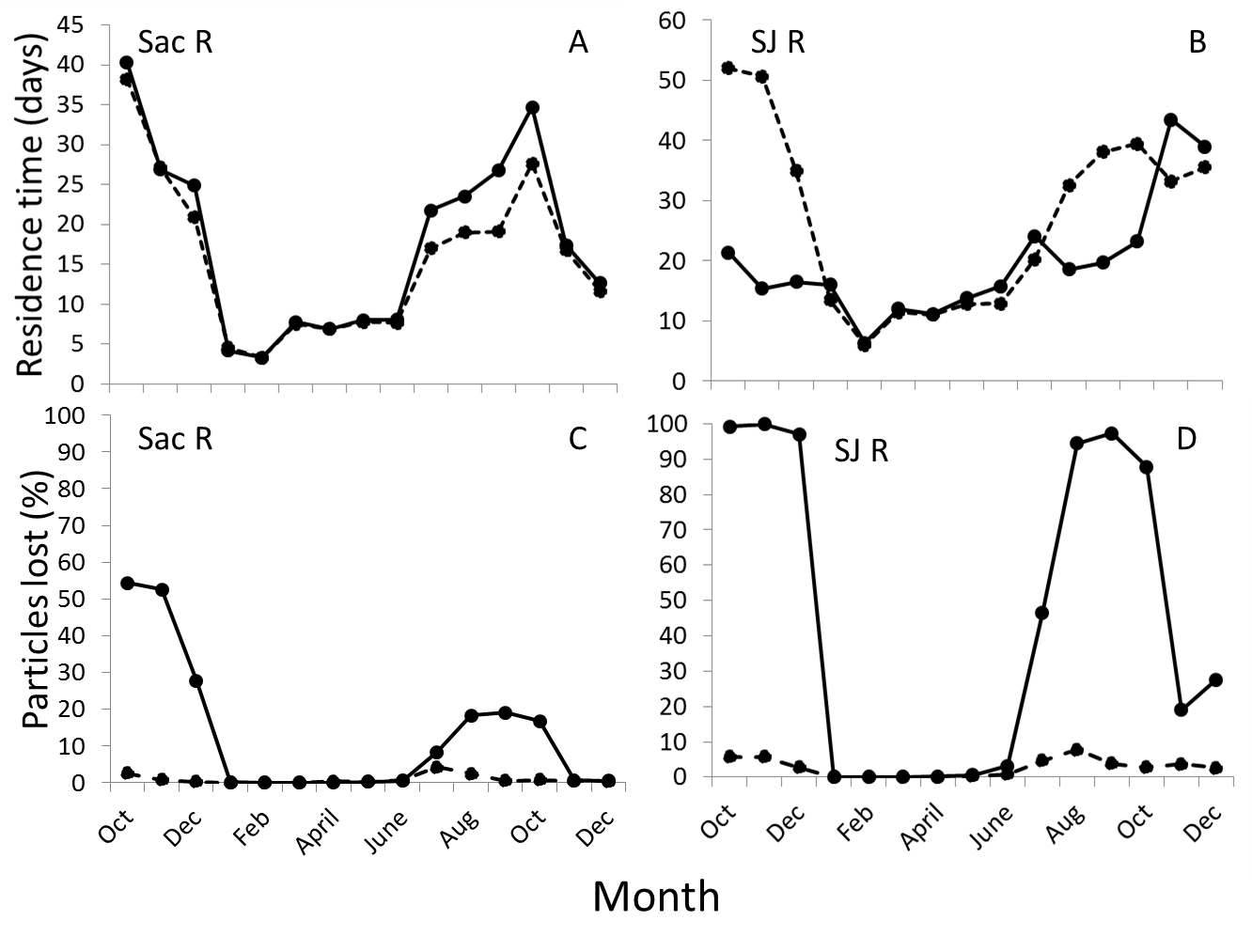


Fig. S10 Residence time (panels A and B) and particles lost (%; panels C and D) with and without historical levels of CVP and SWP pumping for the Sacramento River (panels A and C) and Joaquin River (panels B and D). Solid lines are ‘pumping’ and dashed lines are ‘no pumping’. The plot includes water year 1998 (a period of extremely high outflow) through the first three months of water year 1999. Particles were released on the 15^th^ of each month.


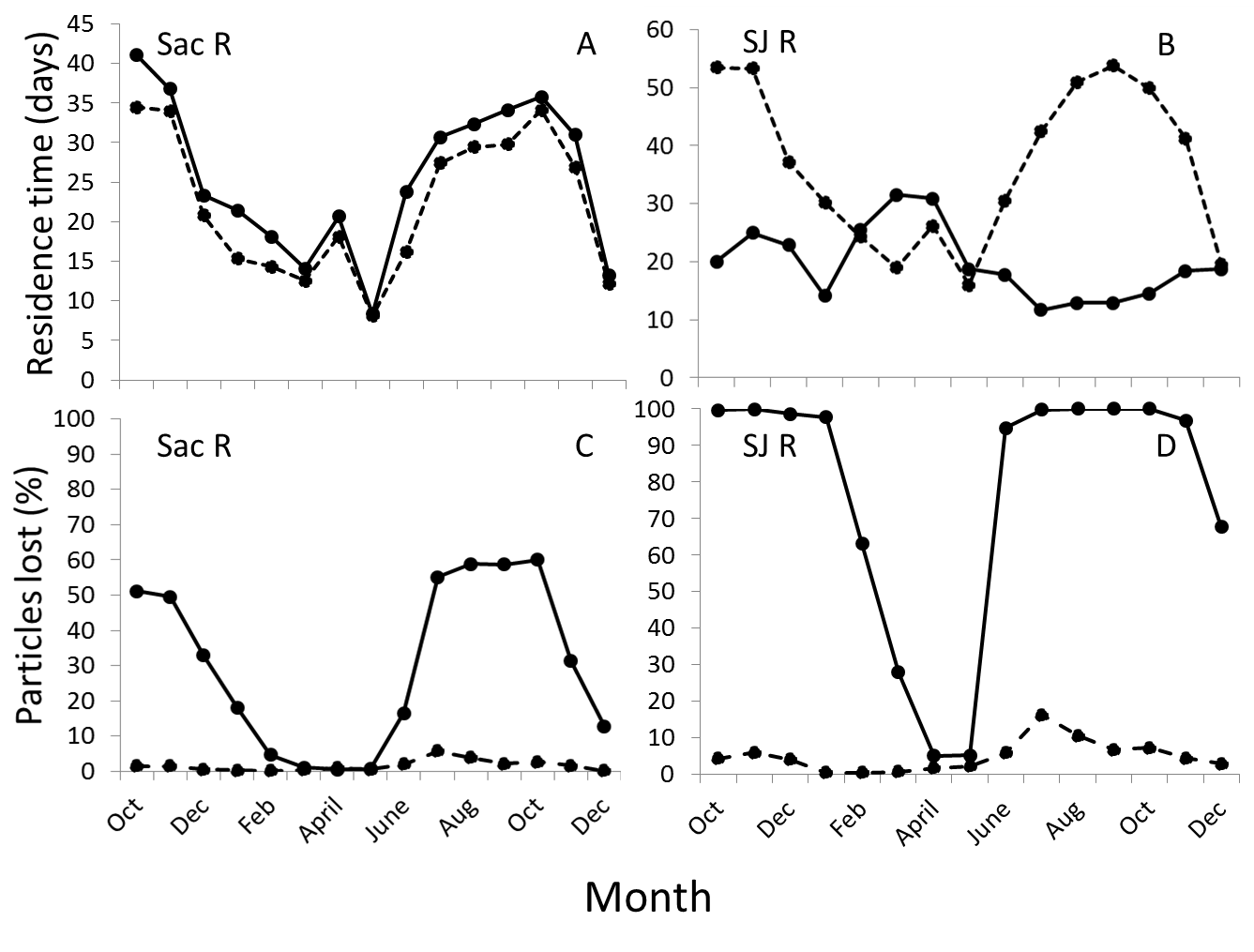


Fig. S11 Residence time (panels A and B) and particles lost (%; panels C and D) with and without historical levels of CVP and SWP pumping for the Sacramento River (panels A and C) and Joaquin River (panels B and D). Solid lines are ‘pumping’ and dashed lines are ‘no pumping’. The plot includes water year 2005 (a period of above average outflow) through the first three months of water year 2006. Particles were released on the 15^th^ of each month.


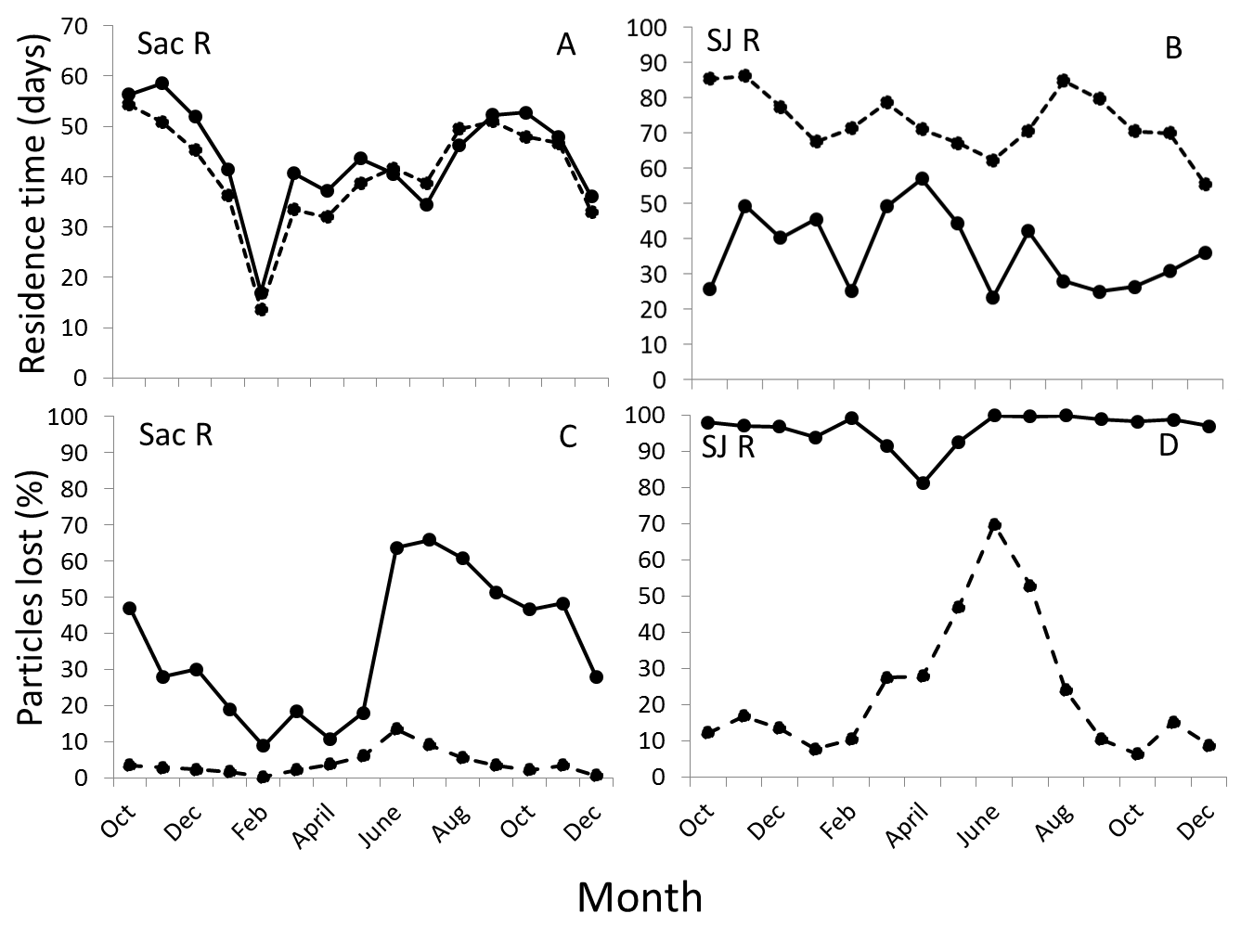


Fig. S12 Residence time (panels A and B) and particles lost (%; panels C and D) with and without historical levels of CVP and SWP pumping for the Sacramento River (panels A and C) and Joaquin River (panels B and D). Solid lines are ‘pumping’ and dashed lines are ‘no pumping’. The plot includes water year 2009 (a period of below average outflow) through the first three months of water year 2010. Particles were released on the 15^th^ of each month.


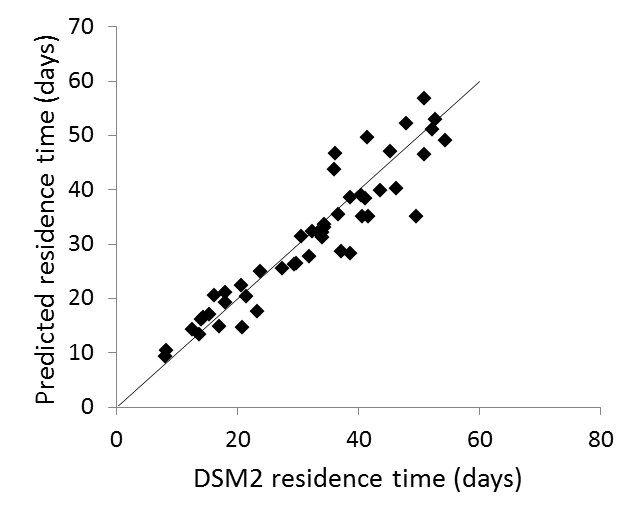


Fig. S13 Validation of the statistical model used to predict residence time on the Sacramento River. The line represents a 1:1 relationship between DSM2 results and statistical model predictions. Water years 2005 and 2009 were used for the validation, with and without the CVP and SWP pumping.


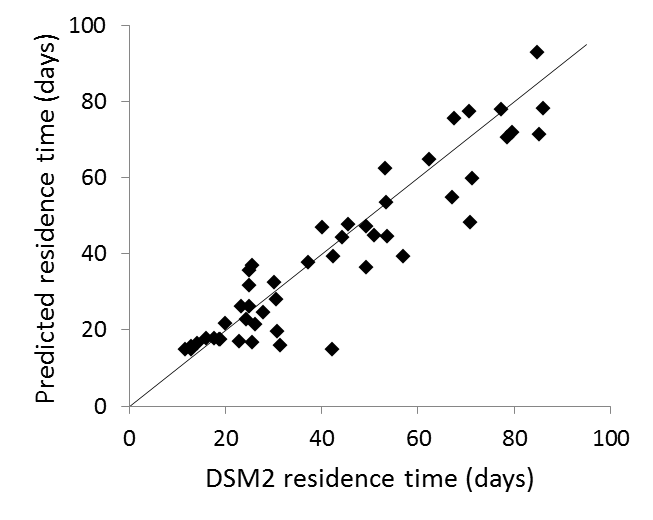


Fig. S14 Validation of the statistical model used to predict residence time on the San Joaquin River. The line represents a 1:1 relationship between DSM2 results and statistical model predictions. Water years 1996 and 2009 were used for the validation, with and without CVP and SWP pumping.


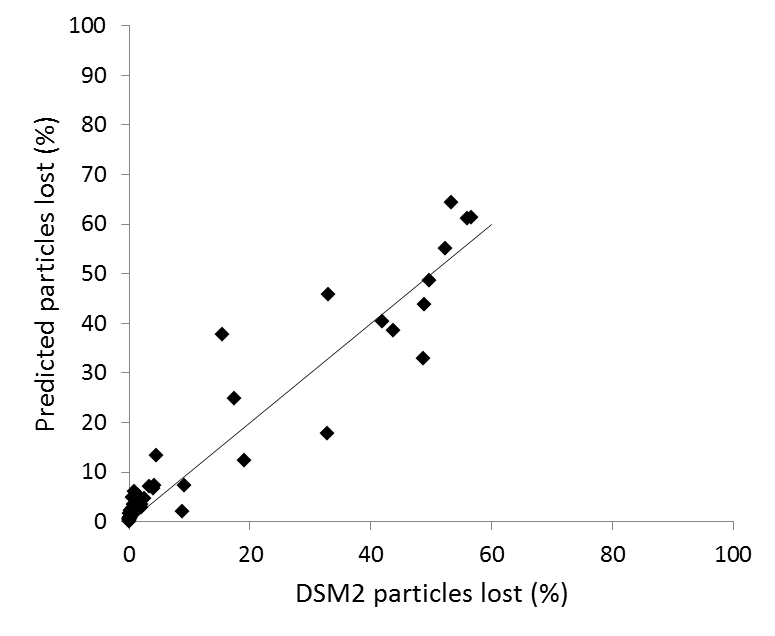


Fig. S15 Validation of the statistical model used to predict particles lost to the CVP, SWP, or agricultural diversions released on the Sacramento River. The line represents a 1:1 relationship between DSM2 results and the statistical model predictions. Water years 1996 and 2009 were used for the validation, with and without CVP and SWP pumping.


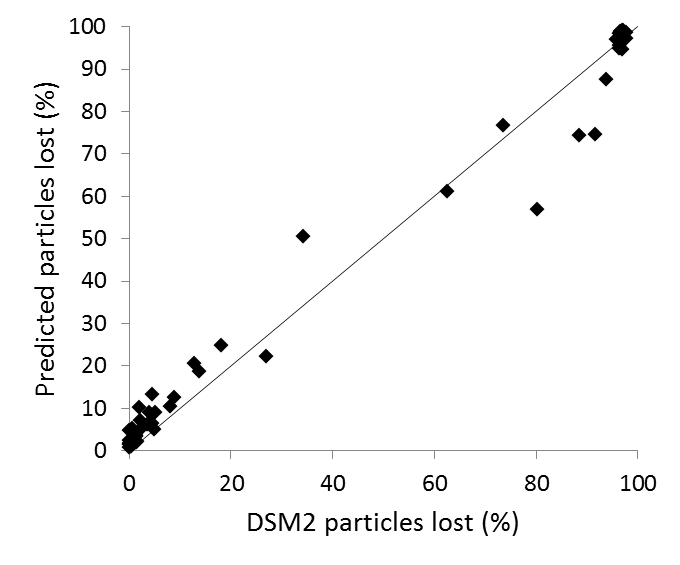


Fig. S16 Validation of the statistical model used to predict particles lost to the CVP, SWP, or agricultural diversions released on the San Joaquin River. The line represents a 1:1 relationship between DSM2 results and the statistical model predictions. Water years 1996 and 2009 were used for the validation, with and without CVP and SWP pumping.


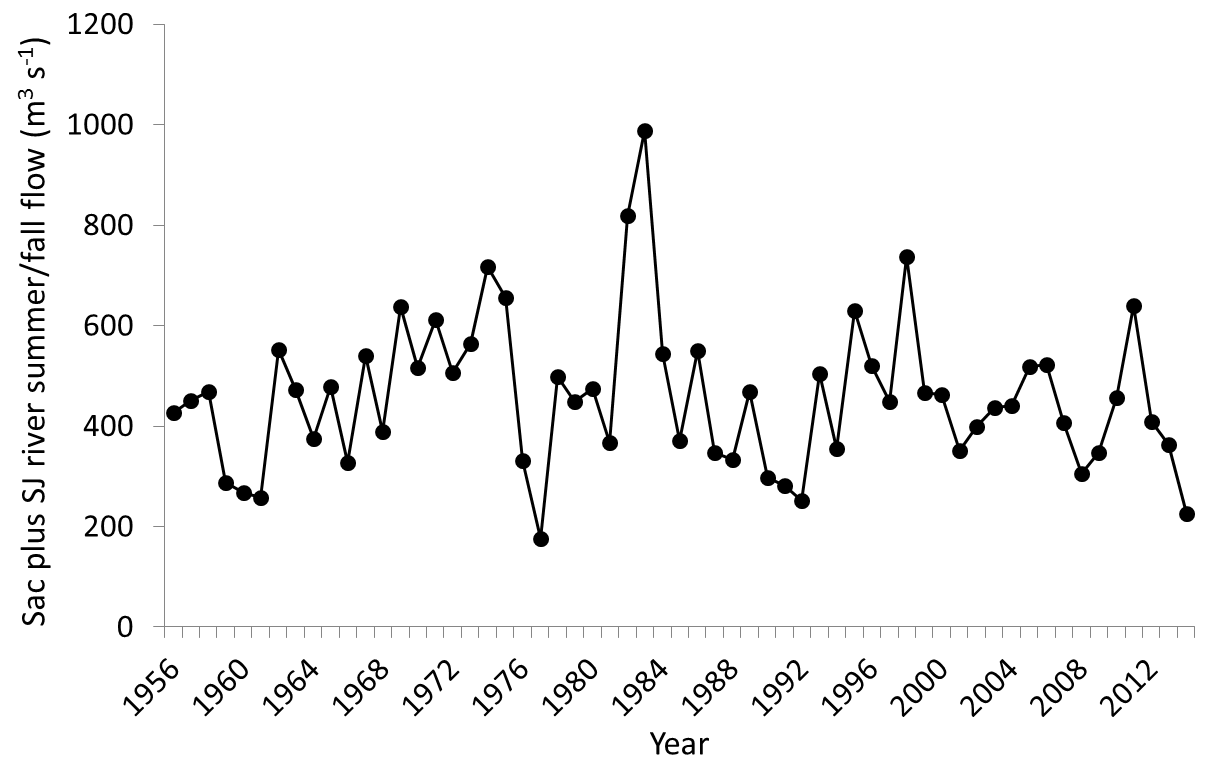


Fig. S17 Mean flow (m^3^ s^-1^) during Aug, Sep, and Oct on the Sacramento plus San Joaquin rivers by calendar year.


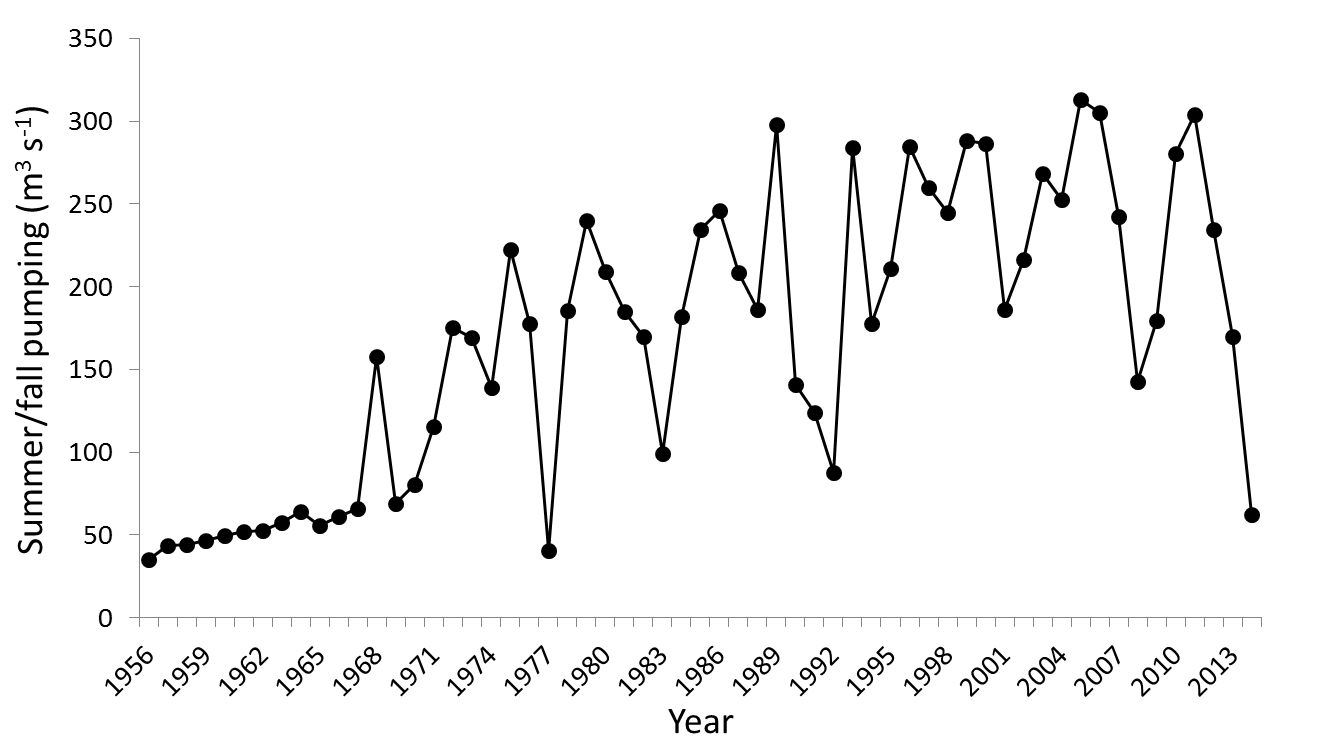


Fig. S18 CVP plus SWP pumping (m^3^ s^-1^) by calendar year, averaged over Aug, Sept, and Oct.


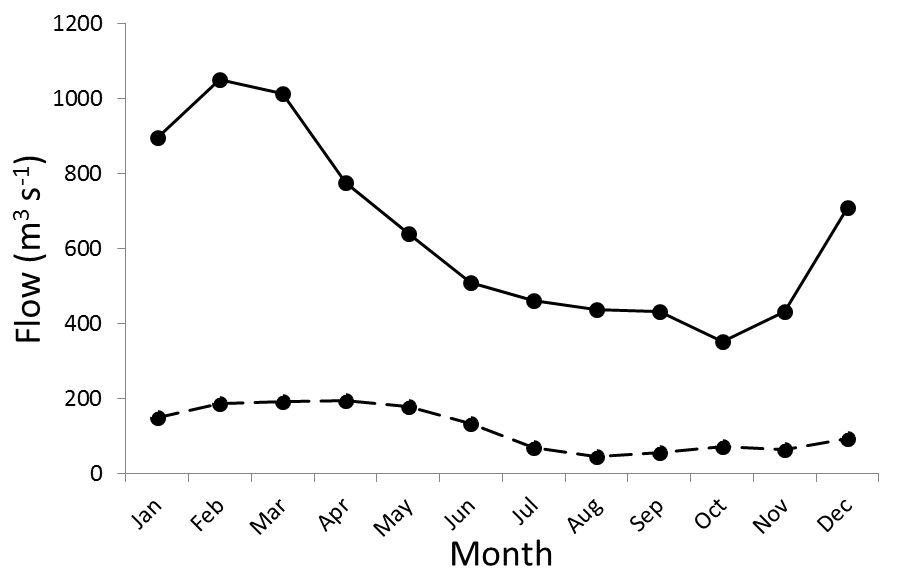


Fig. S19 Mean monthly flow (m^3^ s^-1^) for the Sacramento (solid line) and San Joaquin (dashed line) rivers. Calculations made from 1956-2014 water years.


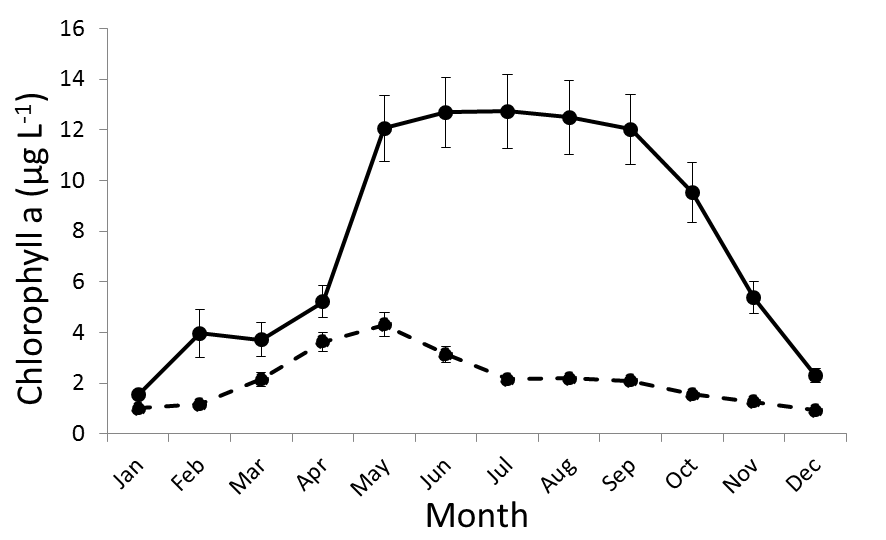


Fig. S20 Mean chlorophyll a by month before and after 1986, averaged across three stations in the SFE (D7, D8, and D10). The period before 1986 (1975-1986) is characterized by reduced water exports and it predates the invasion of *P. amurensis* (solid line)*.* The period after 1986 (1987-2014) has increased water exports and *P. amurensis* is present (dashed line). Error bars are ±SE.

Table S1 Sacramento and San Joaquin river residence time model comparison. Flow is mean flow from the 15^th^ of each month to the 14^th^ of the next month on the Sacramento River at Freeport or on the San Joaquin River at Vernalis. Ag is agricultural diversions, Pump is CVP plus SWP pumping, and NMFlow is the mean flow for the following month (also the 15^th^-14^th^). Residence time and flow were log_10_-transformed.

| Sacramento River residence time | | | | |
| --- | --- | --- | --- | --- |
| Model # | Model | ΔAIC_c_ | df | AIC_c_ wt |
| 7 | ~Flow + Ag + Pump + Ag × Flow | 0.0 | 6 | 0.3907 |
| 9 | ~Flow + Ag + Pump + Ag × Flow + NMFlow | 0.7 | 8 | 0.2800 |
| 8 | ~Flow + Ag + Pump + Ag × Flow + Flow × Pump + NMFlow | 0.9 | 7 | 0.2433 |
| 6 | ~Flow + Ag + Pump + Flow × Pump | 3.2 | 6 | 0.0796 |
| 5 | ~Flow + Pump + Flow × Pump | 9.7 | 5 | 0.0031 |
| 4 | ~Flow + Pump | 10.7 | 4 | 0.0018 |
| 10 | ~Flow + Pump + Flow × Pump + Ag + Ag × Flow + NMFlow | 11.3 | 5 | 0.0014 |
| 2 | ~Flow | 18.8 | 3 | <0.001 |
| 1 | ~Intercept | 342.6 | 2 | <0.001 |
| 3 | ~Pump | 344.3 | 3 | <0.001 |
| San Joaquin River residence time | | | | |
| 7 | ~Flow + Pump + Flow × Pump + Ag + Flow × Ag | 0.0 | 7 | 0.65 |
| 8 | ~Flow + Pump + Flow × Pump + Ag + Flow × Ag + NMFlow | 1.2 | 8 | 0.35 |
| 6 | ~Flow + Pump + Flow × Pump + Ag | 20.5 | 6 | <0.001 |
| 5 | ~Flow + Pump + Flow × Pump | 26.0 | 5 | <0.001 |
| 4 | ~Flow + Pump | 72.5 | 4 | <0.001 |
| 2 | ~Flow | 163.5 | 3 | <0.001 |
| 3 | ~Pump | 227.0 | 3 | <0.001 |
| 1 | ~Intercept | 263.2 | 2 | <0.001 |

*ΔAIC_c_* difference between model of interest and top-ranked model in Akaike Information Criterion Units corrected for small sample size, *df* degrees of freedom, *AIC_c_ wt* Akaike weight

Table S2 Sacramento and San Joaquin river particle fate model comparison. The response variable is ‘proportion of particles lost to agricultural diversions or CVP plus SWP pumping’. Flow is mean flow from the 15^th^ of each month to the 14^th^ of the next month on the Sacramento River at Freeport or on the San Joaquin River at Vernalis. Ag is agricultural diversions and Pump is CVP plus SWP pumping. Flow was log_10_-transformed.

| Sacramento River particle fate | | | | |
| --- | --- | --- | --- | --- |
| Model # | Model | ΔAIC_c_ | df | AIC_c_ wt |
| 6 | ~Flow + Pump + Ag + Flow × Pump + Pump × Ag | 0.0 | 7 | 1 |
| 5 | ~Flow + Pump + Ag + Flow × Pump | 14.0 | 6 | <0.001 |
| 4 | ~Flow + Pump + Ag | 30.5 | 5 | <0.001 |
| 3 | ~Flow + Pump | 54.8 | 4 | <0.001 |
| 2 | ~Intercept | 190.3 | 3 | <0.001 |
| 1 | ~Flow | 242.5 | 2 | <0.001 |
| San Joaquin River particle fate | | | | |
| 6 | ~Flow + Pump + Ag + Flow × Pump + Ag × Pump | 0.0 | 7 | 1 |
| 5 | ~Flow + Pump + Ag + Flow × Pump | 20.3 | 6 | <0.001 |
| 4 | ~Flow + Pump + Ag + Flow × Pump + Flow × Ag | 48.7 | 7 | <0.001 |
| 3 | ~Flow + Pump | 59.4 | 4 | <0.001 |
| 2 | ~Flow | 232.5 | 3 | <0.001 |
| 1 | ~Intercept | 275.0 | 2 | <0.001 |

*ΔAIC_c_* difference between model of interest and top-ranked model in Akaike Information Criterion Units corrected for small sample size, *df* degrees of freedom, *AIC_c_ wt* Akaike weight

Table S3 Metrics of model validation for the residence time and particle fate statistical models (Moriasi et al. 2007). Sac is the Sacramento River, SJ R is the San Joaquin River.

|  | Residence time | | Particle fate | |
| --- | --- | --- | --- | --- |
| Metric | Sac | SJ R | Sac | SJ R |
| Coefficient of Efficiency | 0.867 | 0.846 | 0.905 | 0.978 |
| Index of Agreement | 0.965 | 0.959 | 0.976 | 0.994 |
| R^2^ | 0.874 | 0.861 | 0.913 | 0.981 |

Table S4 Parameter estimates, effect sizes, and 95% confidence intervals (CI) for the top-ranked Sacramento and San Joaquin river residence time models.

| Sacramento River residence time | | | | |
| --- | --- | --- | --- | --- |
| Variable | Parameter estimate | | | 95% CI |
| Intercept | 4.121000 | | | 3.96, 4.29 |
| Flow | -1.012000 | | | -1.07, -0.95 |
| Ag div | 0.005748 | | | 0.001, 0.010 |
| Pump | 0.000271 | | | 0.0001, 0.0004 |
| Ag div × Flow | -0.001951 | | | -0.004, -0.0003 |
|  | Model estimates | | |  |
| Variable | Min | | | Max |
| Flow | 73.7 | | | 5.1 |
| Ag div | 25.7 | | | 30.3 |
| Pump | 25.6 | | | 31.3 |
| Ag div × Flow |  | | |  |
| Ag div | 66.5 (min flow) | | | 102.3 (min flow) |
| Ag div | 5.5 (max flow) | | | 4.2 (max flow) |
| San Joaquin River residence time | | | | |
| Variable | | Parameter estimate | 95% CI | |
| Intercept | | 2.9227944 | 2.81, 3.04 | |
| Flow | | -0.6820884 | -0.741, -0.623 | |
| Ag div | | -0.0044666 | -0.006, -0.003 | |
| Pump | | -0.0050784 | -0.006, -0.004 | |
| Pump × Flow | | 0.0019711 | 0.001, 0.002 | |
| Ag div × Flow | | 0.0022309 | 0.001, 0.003 | |
| Model estimates | | | | |
| Variable | | Min | | Max |
| Flow | | 66.8 | | 11.8 |
| Ag div | | 35.5 | | 31.2 |
| Pump | | 45.8 | | 15.5 |
| Pump × Flow | |  | |  |
| Pump | | 114.1 (min flow) | | 14.9 (min flow) |
| Pump | | 10.5 (max flow) | | 16.6 (max flow) |
| Ag div × Flow | |  | |  |
| Ag div | | 77.6 (min flow) | | 41.5 (min flow) |
| Ag div | | 10.1 (max flow) | | 19.7 (max flow) |

Table S5 Parameter estimates, effect sizes, and 95% confidence intervals (CI) for the top-ranked Sacramento and San Joaquin river particle fate models.

| Sacramento River particle fate | | | | |
| --- | --- | --- | --- | --- |
| Variable | Parameter estimate | | 95% CI | |
| Intercept | 5.9297398 | | 3.61, 8.24 | |
| Flow | -3.8123929 | | -4.70, -2.92 | |
| Ag div | 0.0170571 | | 0.01, 0.02 | |
| Pump | 0.0418152 | | 0.02, 0.06 | |
| Pump × Flow | -0.010023296 | | -0.00168, -0.0033 | |
| Ag div × Pump | -0.00005342 | | -0.0001, 0.0000 | |
| Model estimates | | | | |
| Variable | | Min | | Max |
| Flow | | 0.3466 | | 0.0036 |
| Ag div | | 0.0420 | | 0.3440 |
| Pump | | 0.0225 | | 0.7206 |
| Pump × Flow | |  | |  |
| Pump | | 0.0982 (min flow) | | 0.9783 (min flow) |
| Pump | | 0.0018 (max flow) | | 0.0240 (max flow) |
| Ag div × Pump | |  | |  |
| Ag div | | 0.2262 (min pump) | | 0.7781 (min pump) |
| Ag div | | 0.0020 (max pump) | | 0.0233 (max pump) |
| San Joaquin River particle fate | | | | |
| Variable | Parameter estimate | | 95% CI | |
| Intercept | 0.96493730 | | 0.12, 1.81 | |
| Flow | -2.25853420 | | -2.72, -1.80 | |
| Ag div | 0.01664350 | | 0.01, 0.02 | |
| Pump | 0.05129110 | | 0.04, 0.06 | |
| Pump × Flow | -0.01384713 | | -0.0180, -0.0097 | |
| Ag div × Pump | -0.00008334 | | -0.0001, -0.0001 | |
| Model estimates | | | | |
| Variable | Min | | | Max |
| Flow | 0.8321 | | | 0.0137 |
| Ag div | 0.2711 | | | 0.6046 |
| Pump | 0.0701 | | | 0.9917 |
| Pump × Flow |  | | |  |
| Pump | 0.2498 (min flow) | | | 0.9999 (min flow) |
| Pump | 0.0068 (max flow) | | | 0.0905 (max flow) |
| Ag div × Pump |  | | |  |
| Ag div | 0.0403 (min pump) | | | 0.3255 (min pump) |
| Ag div | 0.9941 (max pump) | | | 0.9749 (max pump) |

Table S6 Relationship between sampling locations used in this paper, their names in the original datasets, locations, and sampling seasons. ‘Agency’ is the agency that collected the data (CDFW is California Department of Fish and Wildlife and DWR is California Department of Water Resources). ‘Site in dataset’ is the name of the site in the original dataset, ‘Site on map’ is the name of the site in Fig. 1.

| Agency | Dataset | Sampled | Site in dataset | Site on map | Latitude | Longitude |
| --- | --- | --- | --- | --- | --- | --- |
| CDFW | Mesozooplankton | Mar-Nov | NZ054 | D10 | 38.04224 | -121.89733 |
| CDFW | Mesozooplankton | Mar-Nov | NZ048 | D8 | 38.05907 | -121.95875 |
| CDFW | Mesozooplankton | Mar-Nov | NZ028 | D7 | 38.11716 | -122.02648 |
| CDFW | Fish (FMWT) | Sept-Dec | 509 | D10 | 38.04932 | -121.91456 |
| CDFW | Fish (FMWT) | Sept-Dec | 504 | D8 | 38.06098 | -121.98351 |
| CDFW | Fish (FMWT) | Sept-Dec | 603 | D7 | 38.11391 | -122.05221 |
| DWR | EMP Discrete Water Quality | year-round | D10 | D10 | 38.04683 | -121.92000 |
| DWR | EMP Discrete Water Quality | year-round | D8 | D8 | 38.05773 | -121.99104 |
| DWR | EMP Discrete Water Quality | year-round | D7 | D7 | 38.11714 | -122.04000 |
| DWR | EMP Benthic Monitoring | year-round | D7-C | D7 | 38.11714 | -122.04000 |

References

DSM2PWT. 2001. Enhanced Calibration and Validation of DSM2 HYDRO and QUAL, Draft Final Report, Interagency Ecological Program for the Sacramento-San Joaquin Estuary. November. Sacramento, California.

DWR. 1997. 18th Annual progress report DSM2. June. Sacramento, California. California Department of Water Resources.

DWR. 2009. DSM2 Recalibration. Report prepared by CH2MHill. http://baydeltaoffice.water.ca.gov/downloads/DSM2_Users_Group/BDCP/DSM2_Recalibration_102709_doc.pdf California Department of Water Resources.

DWR. 2013. DSM2 version 8.1 Calibration. Memorandum. Sacramento, California. https://dsm2ug.water.ca.gov/documents/18/163187/Memo_DSM2_V8.1Beta_Calibration.docx. California Department of Water Resources.

Liu, L., & Sandhu, P. . 2012. DSM2 Version 8.1 Recalibration (Chapter 3). In Methodology for Flow and Salinity Estimates in the Sacramento-San Joaquin Delta and Suisun Marsh: 33rd Annual Progress Report. Sacramento: California Department of Water Resources, Bay-Delta Office, Delta Modeling Section.

Nicolini, M. H., and D. L. Penry. 2000. Spawning, fertilization, and larval development of *Potamocorbula amurensis* (Mollusca: Bivalvia) from San Francisco Bay, California. Pacific Science **54**:377.

Smith, V. H. 2006. Responses of estuarine and coastal marine phytoplankton to nitrogen and phosphorus enrichment. Limnology and Oceanography **51**:377-384.

Wickham, H. 2016. ggplot2: elegant graphics for data analysis. Springer.
